# A Metal‐Phenolic Nanocluster Orchestrates Mito‐Ca^2+^ Metabolic Autonomy for Tumor Ca^2+^ Interference Therapy

**DOI:** 10.1002/advs.76243

**Published:** 2026-06-22

**Authors:** Ronglong Chen, Jia Huang, Yuepeng Wang, Shucong Yao, Fei Wu, Juan Liu, Chunxue Song, Li Lin, Xijun Lin, Yingjie Deng, Chao Zhang, Zhiquan Huang, Zixian Huang, Lisi Xie

**Affiliations:** ^1^ Guangdong Provincial Key Laboratory of Malignant Tumor Epigenetics and Gene Regulation Medical Research Center Sun Yat‐Sen Memorial Hospital Sun Yat‐Sen University Guangzhou China; ^2^ Reproductive Medicine Center The First Affiliated Hospital Sun Yat‐Sen University Guangzhou China; ^3^ Department of Oral and Maxillofacial Surgery Sun Yat‐sen Memorial Hospital Sun Yat‐Sen University Guangzhou China; ^4^ Nanhai Translational Innovation Center of Precision Immunology Sun Yat‐Sen Memorial Hospital Foshan China; ^5^ Department of Oral and Maxillofacial Surgery Nanfang Hospital Southern Medical University Guangzhou China; ^6^ Department of Musculoskeletal Oncology The First Affiliated Hospital Sun Yat‐Sen University Guangzhou China; ^7^ Beijing Institute of Basic Medical Sciences Beijing China

**Keywords:** H_2_S, metal‐phenolic networks, mitochondrial calcium uniporter modulation, mitochondrial fusion suppression, tumor Ca^2+^ interference therapy

## Abstract

Mitochondrial Ca^2+^(mito‐Ca^2+^) interference, an effective mitochondria destruction strategy in cancer treatment, suffers from precise self‐protective Ca^2+^ metabolic autoregulation by Ca^2+^ channels. Direct regulation of Ca^2+^ channels‐mediated mito‐Ca^2^^+^ metabolic autonomy will overcome this obstacle for advanced Ca^2+^ interference therapy. Here, we engineer a novel meta‐phenolic nanocluster (TCMH) by self‐assembly of polyphenols encapsulating mitochondrial calcium uniporter (MCU) modulators (mitofusin 1 siRNA (siMFN1), H_2_S donor) and Ca^2+^ via metal‐phenolic coordination. After cellular internalization, the acid‐responsive disassembly of TCMH triggers the release of its’ payloads. TCMH promotes a rapid surge in mito‐Ca^2+^ via synergistic MCU activation by siMFN1‐mediated mitochondrial fusion suppression and released H_2_S. Furthermore, integrating with H_2_S‐mediated reactive oxygen species (ROS) enrichment, this Ca^2+^‐based TCMH forms a self‐amplifying ROS‐Ca^2^^+^ positive feedback loop to sensitize mitochondrial apoptosis. TCMH potently benefits robust antitumor therapeutics in both oral squamous cell carcinoma orthotopic and patient‐derived xenograft models. Together, a powerful MCU‐mediated mito‐Ca^2+^ metabolic autonomy modulation tactic is highlighted here to optimize precise tumor Ca^2+^ interference therapy.

## Graphical abstract

Tumor Ca^2+^ interference therapy suffers from self‐protective Ca^2+^ metabolic autoregulation. In this scenario, a versatile metal‐phenolic nanocluster (TCMH) is engineered to modulate mitochondrial calcium uniporter (MCU) ‐mediated mito‐Ca^2+^ metabolic autonomy. By the synergistic MCU activation effect of mitochondrial fusion inhibition and H_2_S, TCMH boosts Ca^2+^ influx and self‐amplifies Ca^2^^+^ overload‐sensitive apoptosis.

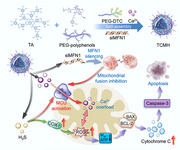

## 1 Introduction

Mitochondrial Ca^2+^(mito‐Ca^2+^) interference therapy, an effective mitochondria destruction strategy, has achieved immense advances against tumor. Frustratingly, tumor has precise self‐protective Ca^2+^ metabolic autoregulation to prevent damage from Ca^2+^ overload [1, 2, 3, 4]. In the context of successful Ca^2+^ delivery by nanocarriers into tumor cells or advanced Ca^2+^ nanomodulators‐mediated elevating mitochondrial Ca^2+^ concentration by stimulating Ca^2+^ release from the endoplasmic reticulum to the cytoplasm and suppression of cell membrane Ca^2+^ efflux [5], aberrantly hyperactive mitochondria constantly sustain ingenious Ca^2^^+^ metabolic autoregulation via Ca^2+^ channel proteins, blocking excessive Ca^2^^+^ influx and promoting excess Ca^2+^ efflux [1, 2, 6]. Direct regulation of mito‐Ca^2^^+^ metabolic autonomy and potentiating the surge in Ca^2^^+^ influx is an important strategy for enhancing mitochondrial Ca^2^^+^ interference therapy [5, 7, 8, 9]. Mitochondrial calcium uniporter (MCU), a highly selective Ca^2+^ channel protein on the inner mitochondrial membrane, governs cytosol‐to‐mitochondrial matrix Ca^2^^+^ influx, making it as a prime autoregulatory target for Ca^2^^+^ uptake [10, 11, 12, 13]. However, although some studies have been reported on MCU regulation‐mediated Ca^2+^ interference therapy, developing effective nanomodulators targeting MCU remains challenging [9, 14, 15, 16]. The presence of multiple membrane barriers, including the plasma membrane, the endoplasmic reticulum membrane, and the outer mitochondrial membrane, severely hampers nano‐modulators’ efficient targeting MCU [17, 18, 19]. Moreover, engineering precise MCU nano‐modulators needs complicate synthetic routes and relatively high costs. Herein, developing a versatile and efficacious MCU‐mediated mito‐Ca^2^^+^ metabolic autonomy regulation strategy will be of profound importance to mitochondria‐targeted Ca^2+^ interference therapy.

Mitochondrial dynamics is closely to mito‐Ca^2^^+^ homeostasis regulation, especially Mitofusins (MFNs)‐mediated mitochondrial fusion [20, 21]. Noted that MFN1 inhibition impairs the mitochondrial fusion process and further leads to MCU activation, priming potent Ca^2^^+^ influx in mitochondria [22, 23, 24]. To fully activate the MCU and attain robust modulation of mitochondrial Ca^2^^+^ metabolism, a combination of mitochondrial fusion inhibition and an alternative MCU regulatory paradigm is imperative. Hydrogen sulfide (H_2_S) can trigger specific mito‐Ca^2+^ surge by opening MCU for cytosolic Ca^2+^ influx via downregulation of MCU gatekeeper, mitochondrial calcium uptake 1 [25]. More importantly, H_2_S can modulate the function of mitochondrial respiratory chain and boost reactive oxygen species (ROS) production [26, 27, 28, 29]. This in turn triggers functional cascade effects including the sustained opening of the mitochondrial permeability transition pore, subsequent increase in the degree of mitochondrial depolarization (ΔΨ m) [1]. This implies that H_2_S may possess the propensity to elicit mitochondrial dysfunction, and cooperation with H_2_S and Ca^2+^ overload‐induced mitochondrial impairment instigate the mitochondrial apoptotic cascade [30, 31]. To date, few studies have addressed the synergistic modulation of mitochondrial fusion inhibition and H_2_S on MCU‐mediated mito‐Ca^2^^+^ metabolic autonomy. Inspired by the aforementioned promising MCU regulative impacts, we propose a sound nexus of mitochondria fusion inhibition and H_2_S with their dominant MCU modulation to realize mito‐Ca^2+^ overload.

**SCHEME 1:**
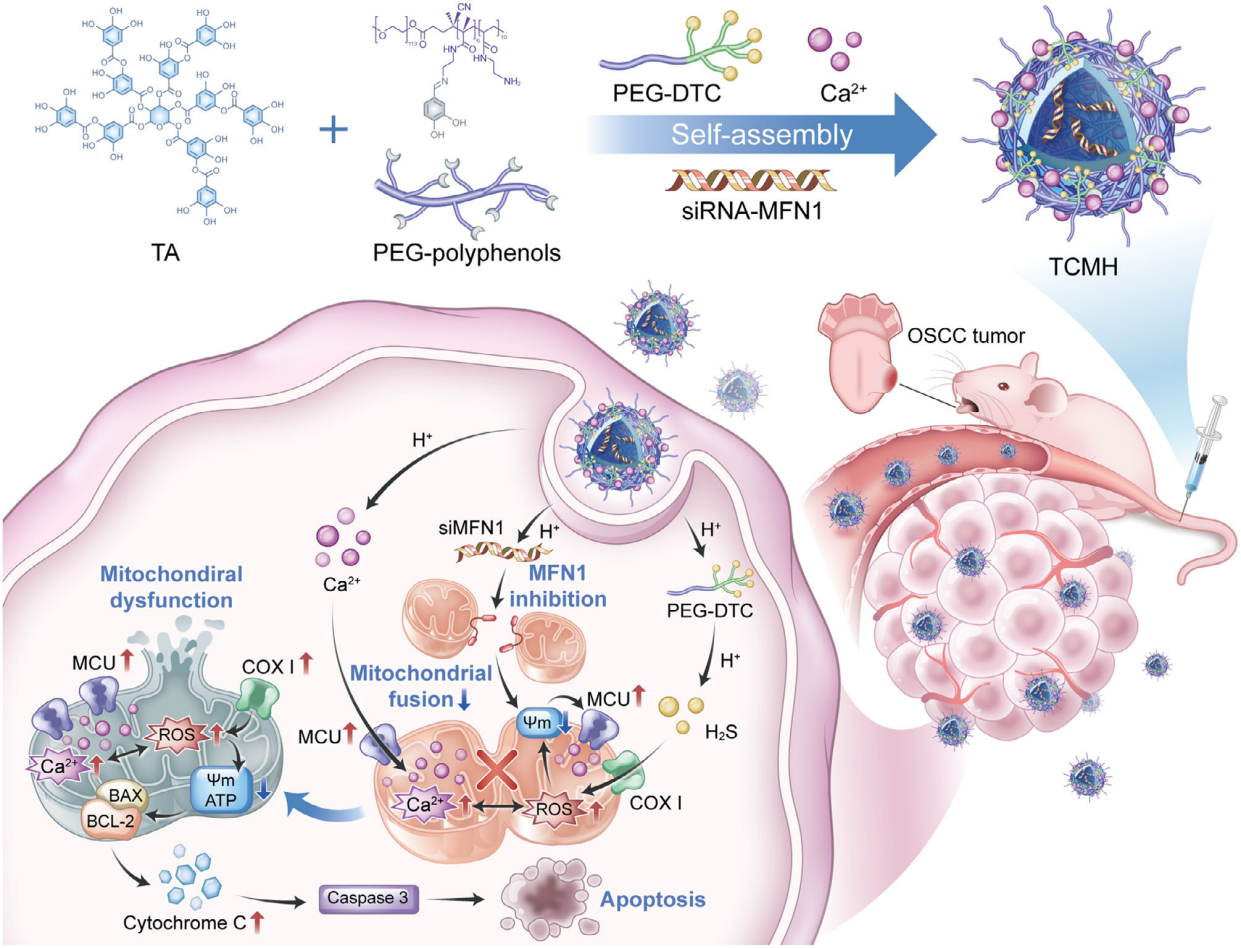
Schematic of TCMH preparation and its role in potentiating tumor Ca^2^^+^ interference therapy via modulating MCU‐based mito‐Ca^2^^+^ metabolic autonomy. First, siMFN1, H_2_S donor (PEG‐DTC), Ca^2^^+^, and polyphenols (TA, PEG‐polyphenols) self‐assembled into a metal‐phenolic nanocluster (TCMH) through metal‐phenolic coordination. Then, after efficient cellular internalization, TCMH achieved acid‐responsive release of its functional components. Specifically, siMFN1 inhibited MFN1 expression to suppress mitochondrial fusion and subsequently initiated MCU activation. Meanwhile, released H_2_S from TCMH synergistically activated MCU, and upregulated COX I for enhancing ROS production. Collectively, TCMH modulated MCU‐mediated mito‐Ca^2+^ metabolic autonomy, thereby promoted Ca^2+^ influx in mitochondrial matrix. TCMH further induced ROS explosion, forming a self‐amplifying ROS‐Ca^2^^+^ positive feedback loop that potently triggered mitochondrial apoptosis. Ultimately, TCMH exerted robust antitumor efficacy.

**FIGURE 1:**
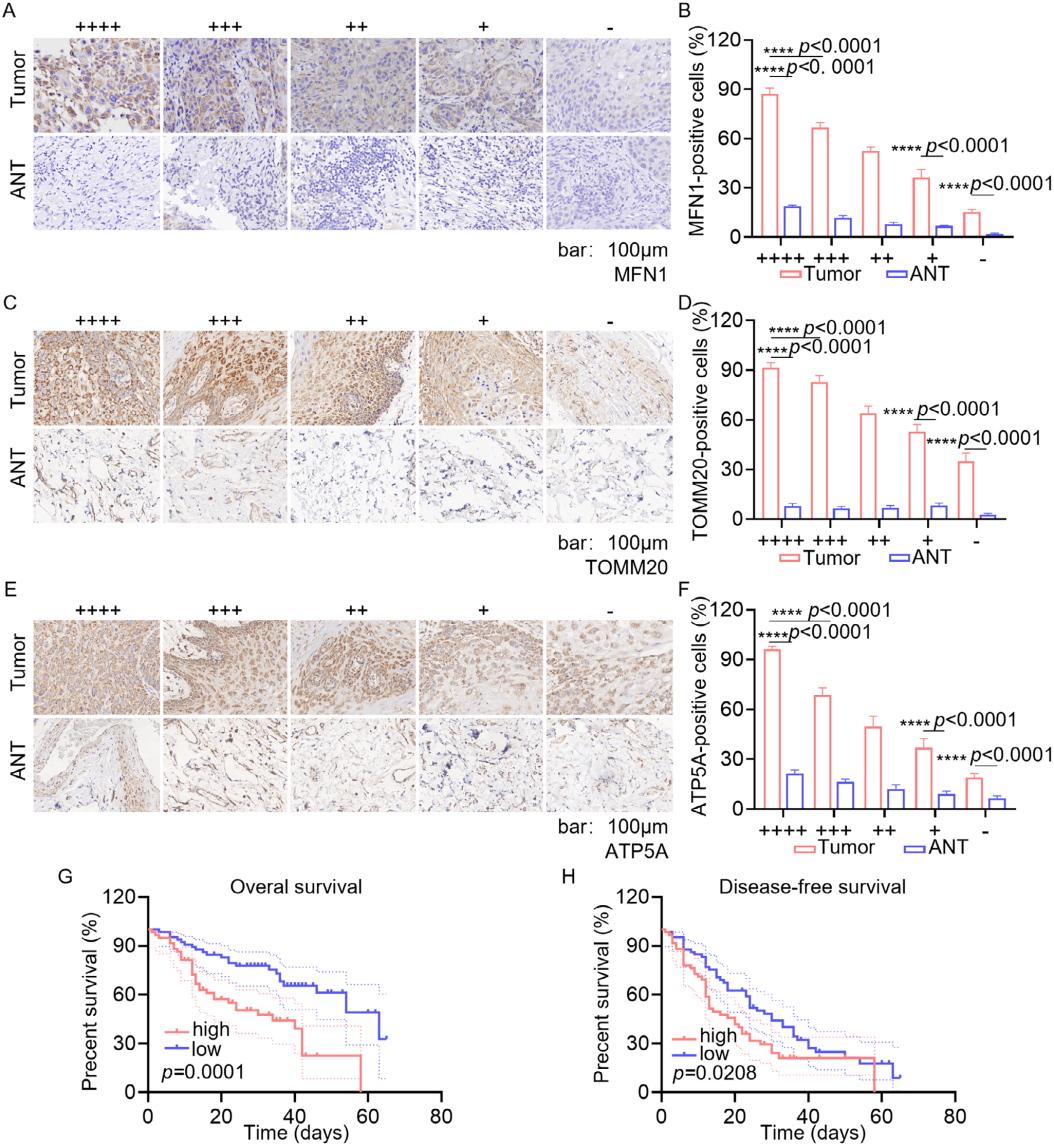
Immunohistochemical analysis of OSCC patient specimens.  ++++: Extremely strong positive, +++: Strong positive, ++: Positive, +: Weakly positive, Adjacent normal tissues: ANT. (A, B) Immunohistochemical detection and quantitative analysis of MFN1. (C, D) Immunohistochemical detection and quantitative analysis of TOMM20. (E, F) Immunohistochemical detection and quantitative analysis of ATP5A. All the sections’ scale was 100 µm. The data were presented as mean ± SD (*n* = 71). (G, H) The overall survival rate (OS) and disease‐free survival rate (DFS) of OSCC patients classified by MFN1 high or low expression level (*n* = 124). The data were presented as mean ± SD (*n* = 124). Statistical significance was calculated via one‐way ANOVA with Tukey's test: *p < 0.05, **p < 0.01, ***p < 0.001, and ****p < 0.0001.

Herein, a metal phenolic nanocluster specifically activating the MCU is engineered here to realize high aggregation of Ca^2+^ in mitochondria matrix for mitochondrial dynamics and function intervention. MFN1‐siRNA (siMFN1) is employed to inhibit MFN1. The hydrophilic nature of siMFN1 drives us to develop metal‐phenolic networks (MPNs) by using polyphenols including tannic acid (TA) and PEG‐modified polyphenols (PEG‐polyphenols) that possess abundant functional ligands to stabilize siRNA encapsulation and long circulation in vivo [32, 33, 34, 35, 36]. In this nano‐system, Ca^2+^ and siMFN1 are coordinated with polyphenols to form siMFN1‐based MPNs (TCM). These amphiphilic MPNs can further carry hydrophilic therapeutic agents via metal‐phenolic coordination, which boasts superior attributes (e.g., strong metal chelation ability, rapid coordination process, and pH responsiveness) of rich phenolic groups [37, 38]. Hence, subsequent encapsulating H_2_S donor (PEG‐DTC) into TCM yielded our functional metal‐phenolic nanocluster (TCMH) (Scheme 1). Notably, clinical sample analysis revealed the high expression of MFN1 exhibited a positive correlation with poor oral squamous cell carcinoma (OSCC) prognosis (Figure 1). This mito‐Ca^2+^ overload strategy will benefit OSCC therapy. Therefore, we used OSCC tumor model in subsequent experiments. The nanocluster showed favorable systematic circulation performance and remarkable delivery performance. Importantly, after tumor accumulation, TCMH enabled siMFN1 delivery to inhibit mitochondrial fusion, thereby it initiated MCU activation and subsequent mitochondrial depolarization, boosting mitochondrial sensitivity to Ca^2+^ overload. Additionally, released H_2_S from TCMH induced MCU activation and mitochondrial dysfunction. Mutually, TCMH modulated MCU‐mediated mito‐Ca^2+^ metabolic autonomy, thereby promoting a rapid surge in mitochondrial Ca^2+^ ions and Ca^2+^ homeostasis imbalance; the latter further boosted ROS production, forming a self‐amplifying ROS‐Ca^2+^ feedback loop that powerfully worsened the mitochondrial dysfunction and then activated the mitochondrial apoptotic pathway. Eventually, TCMH elicited a strong antitumor response in OSCC cell‐line‐derived xenograft (CDX) and patient‐derived xenograft (PDX) models. Overall, a valuable self‐intensified mitochondrial fusion inhibition and H_2_S‐enhanced MCU modulation tactic holds great promise for precise tumor Ca^2+^ interference therapy.

## 2 Results and Discussion

### 2.1 Demonstration of the Potential Benefits of MFN1 Inhibition in OSCC Treatment

To investigate the correlation between MFN1 expression and mitochondrial activity in OSCC, and evaluate its potential as a therapeutic target, we first performed histological analyses in OSCC specimens. Immunohistochemical staining revealed that MFN1 was markedly upregulated in tumor tissues, whereas its expression was minimal in adjacent non‐tumorous tissues (Figures 1A and S1A). As MFN1 served as a core mediator of mitochondrial fusion and tightly governed mitochondrial integrity and metabolic adaptation, we next explored whether its expression correlated with mitochondrial abundance and bioenergetic activity in OSCC [39, 40]. Hence, we evaluated the expression of the mitochondrial mass marker TOMM20 and the oxidative phosphorylation (OXPHOS) marker ATP5A. Notably, the distribution of MFN1‐positive cells closely mirrored that of TOMM20‐positive tumor cells (Figure 1B), suggesting a strong association between MFN1 expression and mitochondrial enrichment. Consistent with this observation, both TOMM20 and ATP5A were significantly elevated in tumor tissues compared with adjacent normal tissues (Figures 1C–F and S1B,C). Furthermore, regions exhibiting high MFN1 expression were frequently accompanied by increased TOMM20 and ATP5A staining, indicating that enhanced mitochondrial fusion was associated with increased mitochondrial content and elevated OXPHOS activity in OSCC cells.

Given the close relationship between MFN1 expression and active mitochondrial metabolism, we subsequently examined its clinical significance in a retrospective cohort of 124 OSCC patients. Survival analyses demonstrated that patients with high MFN1 expression exhibited significantly shorter overall survival (OS) and disease‐free survival (DFS) compared with those expressing low levels of MFN1 (Figure 1G,H). These findings indicated that MFN1 was not only a marker of enhanced mitochondrial metabolic activity but also was associated with aggressive tumor behavior and unfavorable clinical outcomes. Conclusively, these compelling findings indicated that targeted inhibition of MFN1 expression in OSCC held substantial potential to sensitize mitochondria‐associated Ca^2+^ interference therapy and augment anti‐tumor efficacy.

### 2.2 Fabrication and Characterization

**FIGURE 2:**
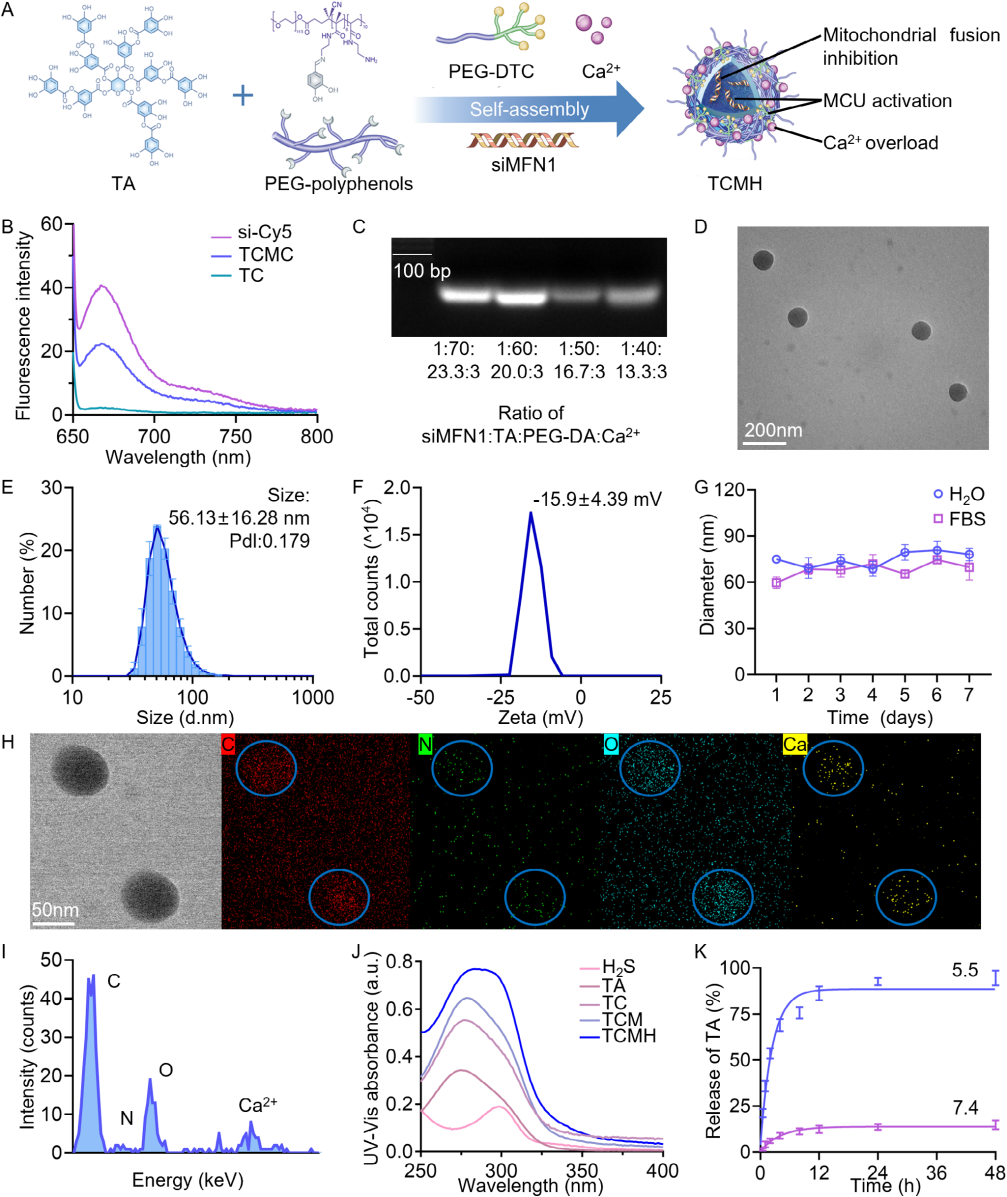
Preparation and characterization of TCMH. (A) The self‐assembly process of TCMH. (B) Fluorescence intensity of si‐Cy5 labeled TCM, TC, and si‐Cy5. (C) DNA gel electrophoresis images under different proportions of TCM NP. (D) Transmission electron microscopy (TEM) images of TCMH. (E) Dynamic light scattering (DLS) data of TCMH. (F) Zeta Potential of TCMH. (G) Diameters of TCMH stored in H_2_O and FBS in 7 days. (H and I) Energy‐dispersive X‐ray spectroscopy (EDS) mapping images and analysis data of TCMH. (J) The UV‐vis absorption spectra of PEG‐DTC, TA, TA chelated with Ca^2+^ (TC), TA coordinated with Ca^2+^, siMFN1 and PEG‐DA (TCM), and TCMH. (K) Percentages of released TA from TCMH over time at different pH values of 5.5 and 7.4.

Inspirated from potent MCU modulation performance of mitofusin 1 (MFN1) downregulation‐associated mitochondria fusion deficiency and H_2_S, a metal‐phenolic nanocluster was fabricated. The self‐assembly process of TCMH was described in the Figure 2A. TA was capable of cross‐linking with siRNA through hydrogen bonding interaction to stable deliver nucleic acids [33, 34, 41]. Thus, TA cross‐linked with siMFN1 was further coordinated with PEG‐polyphenols (PEG‐DA) and Ca^2+^, forming the TA/Ca^2+^‐siMFN1 MPNs (abbreviated as TCM). To further optimize the high loading efficiency of siMFN1 within TCM‐, we fabricated TCM with varying component ratios (Table S1). The siRNA encapsulation efficiency of TCM was further verified by fluorescence spectroscopy, with the negative control TA‐Ca^2+^ MPNs (TC) displaying no fluorescence signal (Figure 2B). A characteristic emission peak around 644 nm of siMFN1‐Cy5 (si‐Cy5) was found, confirming the successful encapsulation of siRNA. The high loading capacity and delivery function of nucleic acids were further tested by an agarose gel electrophoresis assay. The results of fluorescence (FL) intensity and agarose gel electrophoresis collectively confirmed that the siMFN1 encapsulation efficiency reached its maximum value of 79.96% under the optimal mass ratio of 1:60:20:3 (siMFN1: TA: PEG‐DA: Ca^2^^+^) (Figure 2C; Table S1, Figures S2, S3, and S31). The successful synthesis of H_2_S donor (PEG‐DTC) was verified via the nuclear magnetic resonance spectrometer (Figure S4). To further amplify the mitochondrial regulatory efficacy, TCMH were prepared through a self‐assembly approach via coordination interactions among siMFN1, TA/PEG‐DA, PEG‐DTC, and Ca^2+^. The drug loading capacity of TCMH was further optimized (Table S2). In order to optimize the cell growth inhibition, the IC_50_ of PEG‐DTC was first calculated (Figure S5). Then, we prepared the TCMH at a determined concentration of PEG‐DTC (243.9 µg mL^−1^). Excellent calcium loading efficiency (∼61.6%, w/w), and the loading efficiency of si‐Cy5 (∼83.13%, w/w) were achieved at the optimized mass ratio of 1:60:20:1.5:18.8 (siMFN1: TA: PEG‐DA: Ca^2+^: PEG‐DTC, represented by the mass of siMFN1) (Table S2), demonstrating its favorable drug loading performance. Therefore, we ultimately opted to prepare the final product TCMH following the optimized mass ratio of 1:60:20:1.5:18.8, which was a spherical nanomaterial with a diameter of approximately 56.13 ± 16.28 nm, as measured by transmission electron microscopy (TEM) and dynamic light scattering (DLS) (Figure 2D,E). The TCMH had a negative charge of −15.9 ± 4.39 mV, which could benefit its stability in vivo (Figure 2F). The stability of TCMH was further confirmed by monitoring no size change in H_2_O and fetal bovine serum (FBS) over 7 days, verifying the stability and practical feasibility of TCMH in subsequent in vivo experiment (Figure 2G). Ca^2+^ could be successfully chelated with the phenolic groups in TCMH and homogeneously distributed in TCMH, as confirmed by energy dispersive X‐ray spectroscopy (EDS) analysis, high angle annular dark‐field (HAADF) TEM image and corresponding EDS mapping images (Figure 2H,I). TCMH displayed superior Ca iron loading efficiency (∼61.6%, w/w) determined by inductively coupled plasma mass spectrometry (ICP‐MS). Characteristic peaks of TA at 270 nm and H_2_S at 303 nm were observed in TCMH to reveal its successful encapsulation (Figure 2J). Under acidic condition, protonation of phenolic hydroxyl groups on TA and PEG‐DA in TCMH accelerated pH‐dependent disassembly of MPNs and TA release. Accordingly, the pH‐responsive release of TA was confirmed by observed fastest release rate in acidic solution at pH 5.5, indicating the rapid disassembly of TCMH in the cellular acidic environment (Figure 2K). Then, the H_2_S generation efficiency of TCMH was accessed via 5,5'‐dithiobis (2‐nitrophenylacetic acid) (DTNB). At pH 9.0, negligible H_2_S release was detected, indicating the absence of disulfiram. In contrast, at pH 5.5, the cumulative H_2_S release reached approximately 93.6% (1.03 µM) further confirmed the acid‐induced H_2_S release mechanism of the TCMH (Figure S6). Collectively, these findings demonstrate that TCMH represented a promising candidate for further in vitro and in vivo anti‐tumor studies.

### 2.3 In Vitro Endocytosis, Cell Cytotoxicity, and Proliferation Inhibition

**FIGURE 3:**
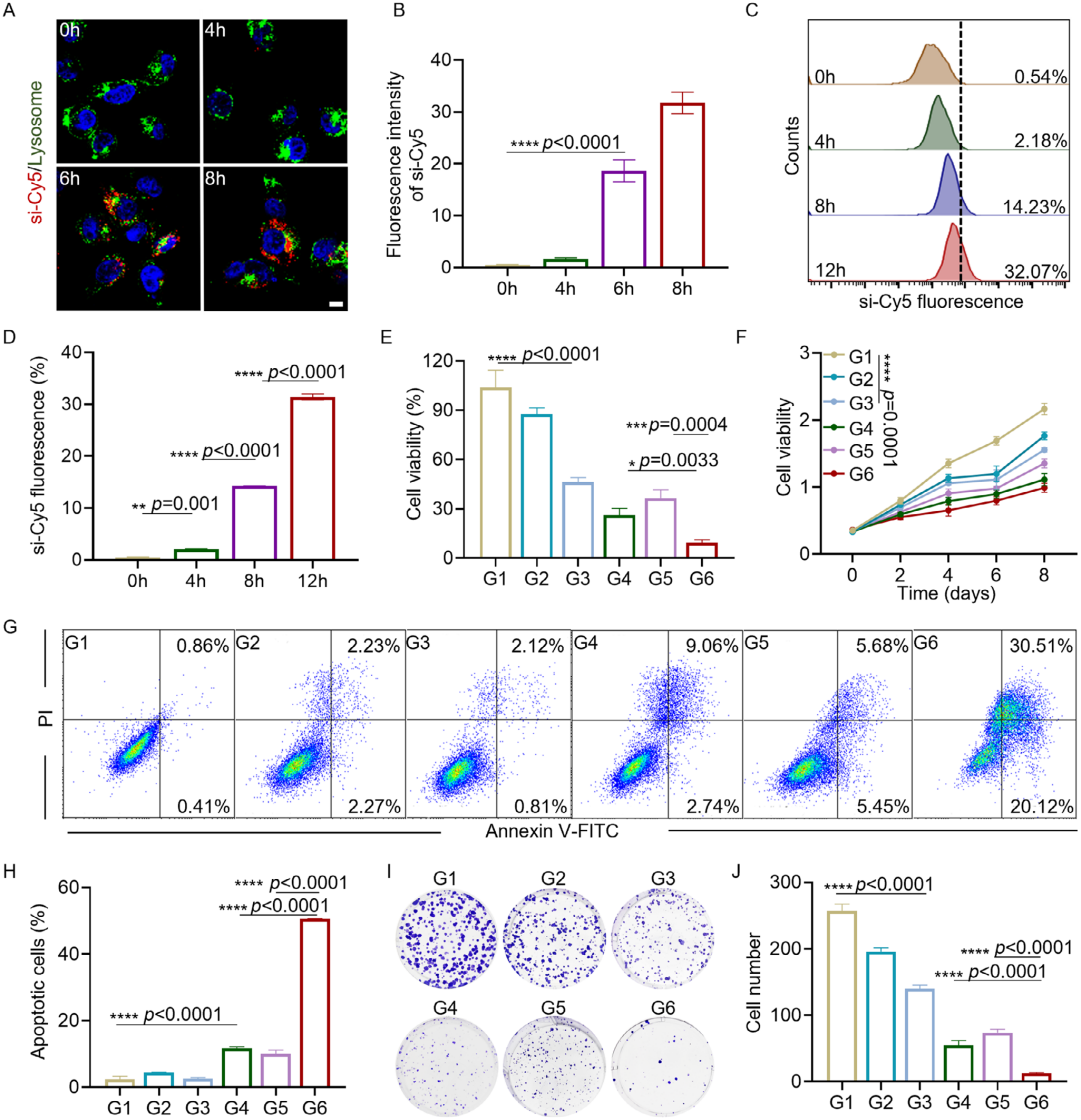
In vitro endocytosis, cell cytotoxicity, and proliferation inhibition. The cells were treated with PBS (G1), TA (G2), TC (G3), TCH (G4), TCM (G5), TCMH (G6) respectively. (A, B) CLSM images and corresponding quantitative analysis of TCMC in Cal‐27 cells. Blue fluorescence represented the nucleus; red fluorescence represented the si‐Cy5; green fluorescence represented the lysosome. The scale bar was 20 µm. (C, D) Flow cytometry images and quantitative analysis of the TCMC in cells. (E) Cell viability of Cal‐27 cells after various treatments for 24 h. (F) Cell growth analysis of Cal‐27 cells after various treatments. (G, H) Apoptosis/necrosis evaluation of Cal‐27 cells after various treatments via flow cytometry and the corresponding quantification analysis. (I, J) Colony formation of Cal‐27 cells after various treatments for 7 days and the corresponding quantitative analysis of colony cell numbers. The data were presented as mean ± SD (*n* = 3). Statistical significance was calculated via one‐way ANOVA with Tukey's test: *p < 0.05, **p < 0.01, ***p < 0.001, and ****p < 0.0001.

Efficient cellular internalization of siRNA carriers profoundly influences transfection efficacy [42, 43]. The endocytosis of TCMH was first evaluated using Cal‐27 cells stably expressing mitochondrial‐targeted GFP (Cal‐27‐mito‐GFP). Here, Cy5‐labeled siMFN1(si‐Cy5) was encapsulated in TA/PEG‐DA/PEG‐DTC‐Ca^2+^ MPNs, designated as TCMC. Confocal laser scanning microscopy (CLSM) and flow cytometry were employed to analyze si‐Cy5 FL intensity in Cal‐27‐mito‐GFP cells treated with TCMC at various time points. TCMC elicited robust cell internalization at 6 h and achieved successful lysosomal escape by 8 h with the evidence of prominent red FL signals of si‐Cy5 detected in Cal‐27 cells (Figures 3A,B and S7). In addition, time‐dependent intercellular uptake behaviors of TCMC in tumor cells were verified via flow cytometry analysis, peaking at 31.39 ± 0.48% at 12 h (Figure 3C,D). Then, the MFN1 silencing efficiency at both RNA and protein levels of TCM was evaluated by quantitative polymerase chain reaction (qPCR) and western blot analysis, respectively. Lipo3000‐transfected siMFN1 was used as a positive control. The result proved that the TCM was similar to the gene silencing efficacy comparable to that of Lipo3000 (Figure S8A). Similar results were found in the MFN1 protein levels (Figure S8B,C). The normal form of mitochondria presents as a long and continuous network structure. However, when mitochondrial fusion is inhibited, the form changes to a scattered and fragmented state [44, 45, 46]. CLSM results revealed that prolonged TCM treatment gradually induced mitochondrial fragmentation in Cal‑27 cells. Accordingly, TCM possessed time‐dependent mitochondria fusion inhibition (Figure S9). Furthermore, both TCM and TCMH groups displayed mitochondria fragmentation analogous to the positive control (Cd^2+^: 500 ng mL^−1^), confirming that MFN1 silencing effectively inhibited mitochondrial fusion (Figure S10). However, MFN1 silencing alone only had a minor effect on tumor cell viability and proliferation, and was insufficient to achieve a significant and sustained anti‐tumor effect (Figure S11).

Mitochondrial apoptosis typically necessitates the synergistic interplay of two or more apoptotic regulatory cues for its effective induction, whereas regulation of mitochondrial fusion via a single modality not only exerts its effects sluggishly but also exhibits insufficient efficacy. In this study, additional mitochondrial regulatory modalities including Ca^2+^ overload and ROS‐mediated oxidative stress responses acted in conjunction with MFN1 silencing would amplify the mitochondrial apoptotic effect. To explore the mechanism of TCMH mediated effective therapeutic effect, cell cytotoxicity, growth, proliferation, and apoptosis were then evaluated. Cell cytotoxicity of Lipo3000/siRNA‐NC, Lipo3000/siMFN1, TA/PEG‐DA chelated with Ca^2+^ MPN (TC), TA/PEG‐DA coordinated with Ca^2+^ and PEG‐DTC MPN (TCH), TCM and TCMH at diverse concentrations was investigated by a CCK‐8 assay. The cells transfected with Lipo3000/siMFN1 exerted a certain inhibitory action on cell viability at concentrations range from 25 to 100 nM, whereas TC exhibited better cytotoxicity effect at high Ca^2+^ concentration range from 30 to 60 µg mL^−1^(Figure S12). TCM showed the combined therapeutic effect of Ca^2+^ and siMFN1 at high concentrations. Besides, introduction of Ca^2+^ and PEG‐DTC in TCH facilitated strong cell toxicity effect with increasing concentrations. Distinctive concentration‐dependent cytotoxicity was found in cells treated with TCMH. Furthermore, at a concentration of 30 µg mL^−1^ Ca^2^^+^, 50 nM siMFN1, and 243.9 µg mL^−1^ PEG‐DTC, TCMH induced approximately 65% cell death (Figure S12). In order to further explore their therapeutic effect, this optimal concentration of TCMH was used for subsequent experiments. The cells were treated with PBS (G1), TA (G2), TC (G3), TCH (G4), TCM (G5), and TCMH (G6). Significantly, the lowest cell viability of 8.75 ± 1.28% was observed in TCMH (G6) (Figure 3E). The cell growth inhibition property of TCMH was confirmed by the OD values for cell viability at 7 days (Figure 3F). An apoptosis/necrosis assay was further conducted to evaluate TCMH‐mediated synergistic antitumor effect. Cells were treated with different nanoformulations and then co‐incubated with fluorescein isothiocyanate (Annexin V‐FITC) and propidium iodide (PI). Early apoptotic cells were stained exclusively with Annexin V‐FITC, while late apoptotic or necrotic cells were co‐stained with Annexin V‐FITC and PI, as analyzed by flow cytometry. TCH (G4) and TCM (G5) induced obvious apoptosis/necrosis‐positive cells of 11.8% and 11.13% respectively (Figure 3G). Compared with other groups, the most significant apoptosis/necrosis‐positive cells (50.59 ± 0.05%) were observed in G6 (Figure 3G,H). Besides, TCMH also shows significant anti‐cancer effects in multiple types of cancers (Figures S13 and S14). Additional cell proliferation property of TCMH were evaluated by colony formation. By virtue of the antitumor effect of Ca^2+^ overload and TA, a slight colonies inhibition effect was found in TC group (G3). By contrast, G4 and G5 showed lower numbers of cell colonies (Figures 3I,J and S15). The lowest colonies (12.3 ± 1.25) were found in G6, illustrating the remarkable cell proliferation inhibition (Figures 3I,J and S15). Collectively, all these promising results suggested that TCMH possessed superior therapeutic efficiency in vitro.

### 2.4 Mitochondrial Function Modulation

**FIGURE 4:**
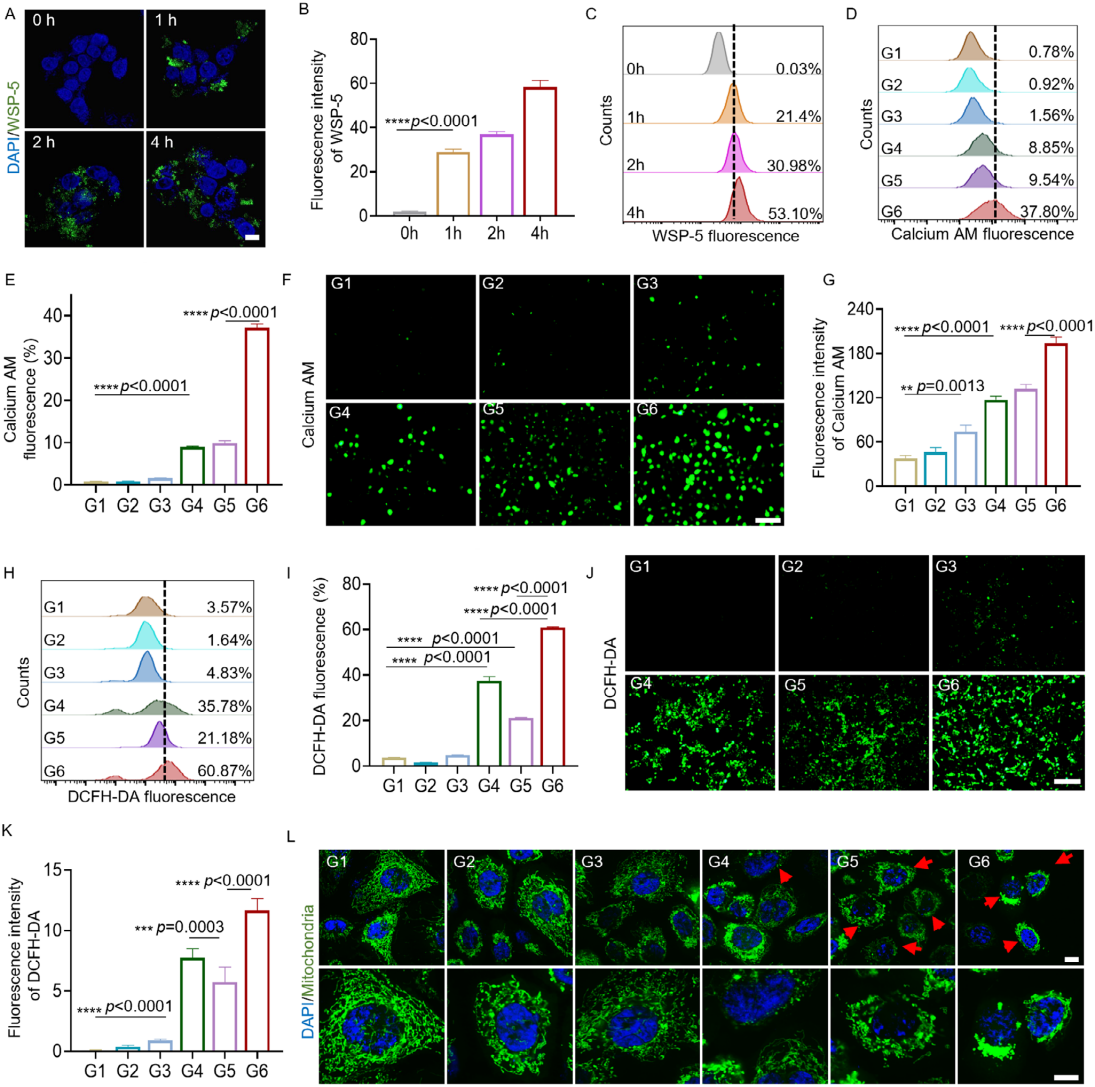
In vitro mitochondrial function regulation. The cells were treated with PBS (G1), TA (G2), TC (G3), TCH (G4), TCM (G5), TCMH (G6). respectively. (A, B) CLSM images and quantitative analysis of H_2_S generation in Cal‐27 cells after incubation with TCMH. Blue fluorescence represented the nucleus; green fluorescence represented the H_2_S detection probe WSP‐5. The scale bar was 20 µm. (C) Flow cytometry analysis of the H_2_S generated from TCM in cells. (D, E) Flow cytometry peak plots and quantitative analysis of Ca^2+^ in the cells treated with TCMH. (F, G) Fluorescence images and quantitative analysis of Ca^2+^ in cells after treatments. Green fluorescence represented the Ca^2+^ detection probe calcium AM. The scale bar was 100 µm. (H, I) Flow cytometry and quantitative analysis of ROS in the cells treated with TCMH. (J, K) Fluorescence images and quantitative analysis of ROS in Cal‐27 cells after 24 h incubation with different nano‐formulas. Green fluorescence represented the ROS detection probe DCFH‐DA. Scale bar was 100 µm. (L) CLSM images of mitochondria change in Cal‐27‐mito‐GFP cells after different treatments. Blue fluorescence represented nucleus; green fluorescence represented mitochondria. The scale bar was 10 µm. The data were presented as mean ± SD (*n* = 3). Statistical significance was calculated via one‐way ANOVA with Tukey's test: *p < 0.05, **p < 0.01, ***p < 0.001, and ****p < 0.0001.

Mitochondrial dysfunction leads to mitochondrial apoptosis, and further promotes cell apoptosis [47, 48]. Previous study has demonstrated that H_2_S and Ca^2+^ overload mediated mitochondrial dysfunction to kill cancer cells [22, 49]. We would further explore the role of TCMH in regulating mitochondrial function. The production of H_2_S was investigated by a WSP‐5 probe through CLSM and flow cytometry analysis. The representative augmented green FL revealed continuous production of H_2_S generation in cells treated with TCMH (Figures 4A–C and S16). Notably, following 4 h of incubation, TCMH elicited the highest levels of intracellular H_2_S (52.2 ± 1.17%) (Figure 4C), indicating its sufficient mitochondrial dysfunction effect. Subsequently, intracellular levels of Ca^2+^ and ROS were detected by 5‐Fluo‐4 M and 2’,7’‐dichlorodihydro‐fluorescein diacetate (H2DCFDA) probes respectively. TC (G3) successfully delivered Ca^2+^ in cells. Intriguingly, TCH (G4) and TCM (G5) generated more Ca^2+^ in cells than G3, suggesting that H_2_S and mitochondrial fusion inhibition might increase Ca^2+^ levels in mitochondria (Figure 4D,E). Integrating H_2_S and siMFN1, TCMH (G6) remarkably triggered the higher level of Ca^2+^ explosion (37.15 ± 0.78%) in cells than G4 (8.99 ± 0.11%) and G5 (9.83 ± 0.49%) (Figure 4E). CLSM images and quantitative analysis further confirmed the highest levels Ca^2+^ generated in G6 (193.78 ± 6.90%) (Figure 4F,G). Similar variance was noted in the ROS generation among the groups corresponded to Ca^2+^ production (Figure 4H). In particular, the ROS levels in G4 and G5 increased by ∼10.02‐fold and ∼5.93‐fold respectively as evidenced by the percentage of FL intensities, indicating that Ca^2+^ increase driven by H_2_S and mitochondria fusion inhibition could promote ROS generation (Figure 4H,I). It is noteworthy that G6 elicited the most pronounced elevation in ROS levels (60.88 ± 0.23%), which unveiled the synergistic interplay among mitochondrial fusion suppression, Ca^2^^+^ overload and H_2_S‐mediated ROS generation (Figure 4I). CLSM images and quantitative analysis also confirmed the highest levels of ROS generated in G6 (9.88 ± 2.75%) (Figure 4J,K). Hence, ROS surge would effectively trigger mitochondrial destruction further. Normal spindle‐shaped mitochondria were observed in the PBS (G1) and TA (G2) groups, whereas a few punctate mitochondria were found in G3, suggesting exogenous Ca^2+^ induced mitochondria morphological change. Besides, elevated levels of Ca^2+^ and H_2_S in G4 triggered synergistic mitochondria dysfunction, thus leading to the formation of more punctate mitochondria compared to the G1‐G3 groups. A large amount of fragmented mitochondria were found in cells treated with G5, indicating the combined mitochondrial fusion inhibition and dysfunction effect of Ca^2+^ overload. TCMH group exhibited the most prominent mitochondrial fragmentation compared to other groups, indicating their potent mitochondria destruction ability (Figure 4L). Definitely, TCMH facilitated strong mitochondria dysfunction as evidence by time‐dependent mitochondria damage (Figure S17). Comprehensively, all these promising results suggested that the biocompatible TCMH possessed notable mitochondrial dysfunction.

### 2.5 MCU‐mediated Mito‐Ca^2+^ Metabolic Autonomy Modulation

**FIGURE 5:**
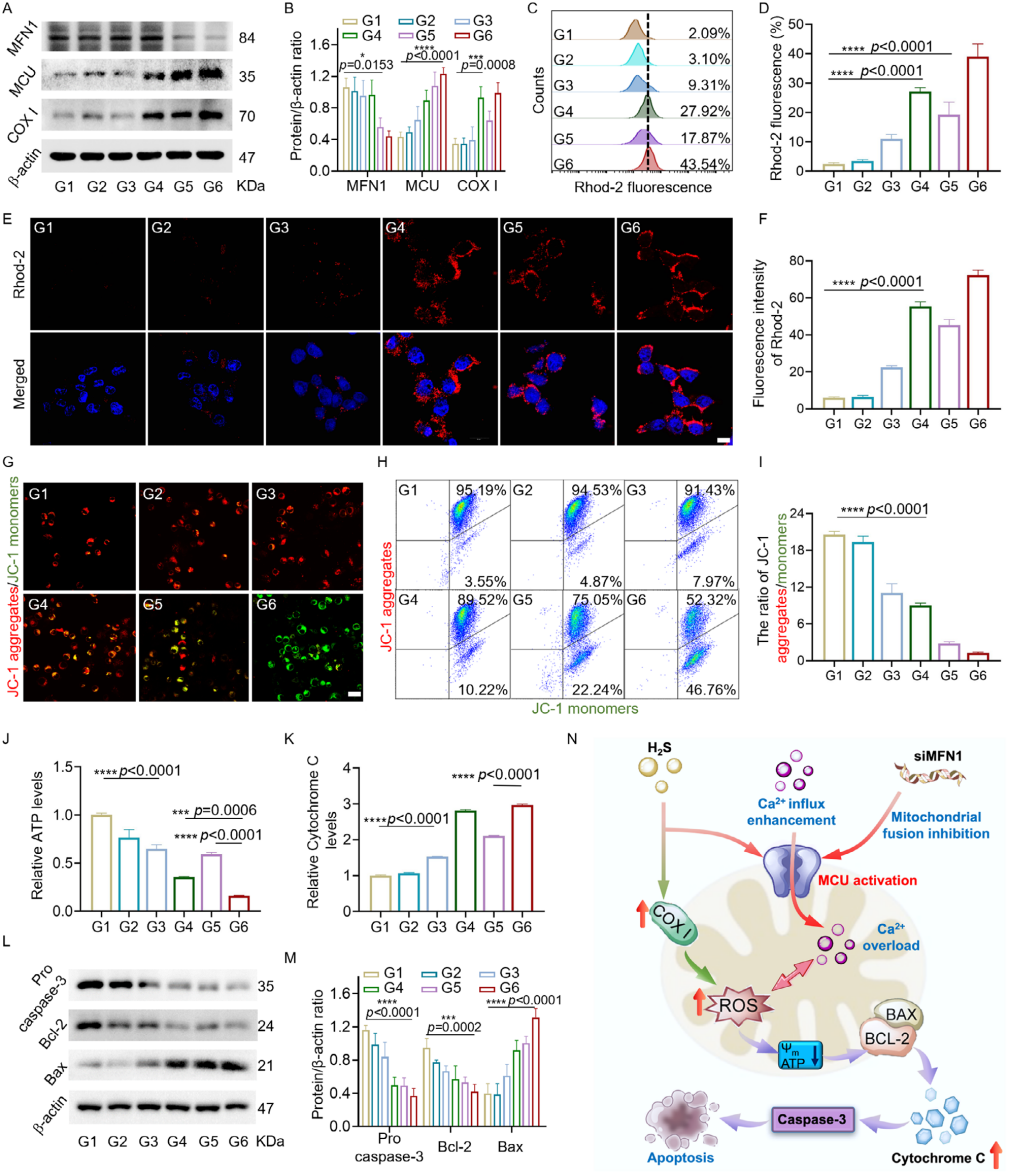
MCU‐mediated mito‐Ca^2+^ metabolic autonomy modulation by TCMH. The cells were treated with PBS (G1), TA (G2), TC (G3), TCH (G4), TCM (G5), TCMH (G6). respectively. (A) Western blot analysis for MFN1, MCU, and COX I proteins. (B) The ratio of gray value of proteins to β‐actin. (C, D) Flow cytometry peak plot and quantitative analysis of mitochondrial Ca^2+^ level detected by Rhod‐2 AM. (E) CLSM images of mitochondrial Ca^2+^ overload. Green FL represented the Ca^2+^ detection probe Rhod‐2 AM. Scale bar was 20 µm. (F) The FL intensity of Rhod‐2. The data of FL was collected from CLSM images. (G) The CLSM images of mitochondria dysfunction in Cal‐27 cells after various treatments, detected by a JC‐1 probe. Red FL represented as health mitochondria, green FL represented as damaged mitochondria. The scale bar was 20 µm. (H, I) Flow cytometry plots graphs and corresponding quantitative analysis of mitochondrial depolarization in Cal‐27 cells after various treatments. (J) The mitochondrial ATP content. (K) The content of cytochrome C. (L) Western blot analysis for apoptosis‐associated proteins. (M) The ratio of gray value of apoptosis‐associated proteins to β‐actin. (N) Schematic mechanism of MCU‐mediated mito‐Ca^2+^ metabolic autonomy modulation by TCMH for mitochondrial apoptosis sensitization. The data were presented as mean ± SD (*n* = 3). Statistical significance was calculated via one‐way ANOVA with Tukey's test: *p < 0.05, **p < 0.01, ***p < 0.001, and ****p < 0.0001.

Evidently, the striking mitochondrial fusion inhibition and H_2_S will contribute to MCU activation for mito‐Ca^2+^ aggregation, benefiting Ca^2+^ overload. Our results indicated that independent H_2_S intervention or siMFN1‐mediated moderate MCU activation only induces slight endogenous Ca^2^^+^ elevation and modest intracellular Ca^2^^+^ accumulation, failing to trigger severe Ca^2+^ overload (Figure S18). When MCU activation combined with exogenous Ca^2+^ supplementation, TCMH amplified mitochondrial Ca^2+^ uptake (Figure S18). Consequently, to further verified the underlying mechanism of TCMH‐elicited MCU‐mediated Mito‐Ca^2+^ metabolic autonomy modulation, a series of experiments including MCU activity, as well as alterations in cytoplasmic Ca^2+^, mitochondrial Ca^2+^, ROS production and mitochondrial apoptosis evaluation were performed. The synergistic MCU modulation of TCMH was re‐investigated. The key proteins including MFN1, MCU, and COX I were further analyzed. G5 and G6 successfully decreased MFN1 protein levels due to their efficient delivery of siMFN1, which would induce MCU upregulation for Ca^2+^ influx so as to ROS production. And marked MCU expression did increase in G5 and G6 (Figures 5A,B and S32). Intriguingly, TCH also increased MCU expression. COX I overactivation could affect the proton pump activity, leading to the disruption of coordination of the respiratory chain to accelerate ROS production [26, 50]. H_2_S ‐mediated high level of COX I upregulation was observed in G4, demonstrating ROS generation ability of H_2_S (Figures 5A,B and S32). Similar results were also observed in the G5 group. It's mainly due to the disruptive effect of mitochondrial fusion inhibition on respiratory chain stability. Distinctively, TCMH elicited more pronounced COX I upregulation than TCH and TCM, reconfirming their robust ROS production ability. To investigated if Ca^2+^ enriched in mitochondria, flow cytometry and CLSM images analysis were performed. Significantly, highest Ca^2+^ level (42.4 ± 3.26%) was detected in G6 (Figure 5C,D). The CLSM images also evidenced that strongest red FL signal of mitochondrial Ca^2+^ probe was detected in G6 (Figures 5E,F and S19). Furthermore, such augmented Ca^2+^ overload serves as a pivotal trigger for ROS detonation for mitochondrial dysfunction and apoptosis sensitization. A JC‐1 probe, the mitochondrial activity detector, was applied to investigate the mitochondrial membrane potential (ΔΨ_m_) change. Cationic JC‐1 probe was attracted by the negative potential of the inner membrane and thus largely enter the mitochondrial matrix. When ΔΨ_m_ decreased or depolarization occurred, the probe would be released from the mitochondria into the cytoplasm, causing characteristic changes in the FL signal in the mitochondrial region. Red FL of J‐aggregates was observed in healthy mitochondria, whereas green FL of J‐monomers represented damaged mitochondria and mitochondrial depolarization (Figures 5G and S20). TA or TC couldn't induce obvious mitochondrial membrane damage. The integration of H_2_S into TCH (G4) modulated mitochondrial depolarization to a moderate extent (Figures 5G and S20). Significant a lower ratio of red FL to green FL was found in TCM (G5) (0.59 ± 0.09) than TC (G3) (5.3 ± 0.22) confirmed the mitochondrial fusion inhibition‐augmented mitochondrial depolarization (Figure S20). Dramatical decrease of red FL with increase of green FL was found in TCMH‐treated cells, suggesting TCMH triggered severe mitochondrial impairment (Figure S20). Such mitochondria depolarization effect was further evidenced by flow cytometry analysis. The percentage of green FL signal of J‐monomers gradually increased (26.29 ± 1.53%), while the J‐aggregates showed a decreasing trend (73.71 ± 1.53%) in G5, which was due to the mitochondrial fusion inhibition mediated mitochondria membrane potential loss (Figure 5H). Significantly, the ratio of the red FL to green FL (∼1.26) is much lower in TCMH group (G6) than TCH group (G4) (∼9.0) and G5 (∼2.82), demonstrating remarkably mitochondrial dysfunction effect with the synergy of siMFN1, Ca^2+^ overload, and H_2_S (Figure 5I).

Next, mitochondrial apoptosis pathway was investigated. Notably, mitochondrial disruption effect of G6 also was confirmed by mitochondrial ATP levels decrease by ∼ 80% (Figure 5J). When mitochondria apoptosis occurred, mitochondrial cytochrome C would release from mitochondria, thereby amplifying the cell apoptosis signal. High level in cytochrome C in G6 increased by ∼2.97‐fold, accelerating caspase‐3 apoptotic pathway activation (Figure 5K). Thus, TCMH exhibited an obvious increase of Bax protein levels alongside a marked decrease in Bcl‐2 protein levels. Meanwhile, the procaspase‑3 protein level decreased gradually, whereas the expression of cleaved caspase‑3 was markedly upregulated. This indicated its’ rich ROS production resulted in effective mitochondrial apoptosis (Figures 5L,M and S21). Collectively, we have preliminarily verified their synergistic mechanism of efficient MCU mediated mito‐Ca^2+^ metabolic autonomy modulation (as illustrated in Figure 5N): TCMH facilitated Ca^2+^ influx enhancement by synergistic MCU activation of siMFN1‐mediated mitochondrial fusion deficiency and H_2_S. Ultimately, TCMH created a positive feedback loop of Ca^2^^+^ overload and ROS production. This MCU‐mediated mito‐Ca^2+^ metabolic autonomy modulation compromised mitochondrial integrity, provoked the release of cytochrome C and activated the caspase‐3 pathway, finally culminating in apoptosis.

### 2.6 Biodistribution and Antitumor Performance in the CDX Model

**FIGURE 6:**
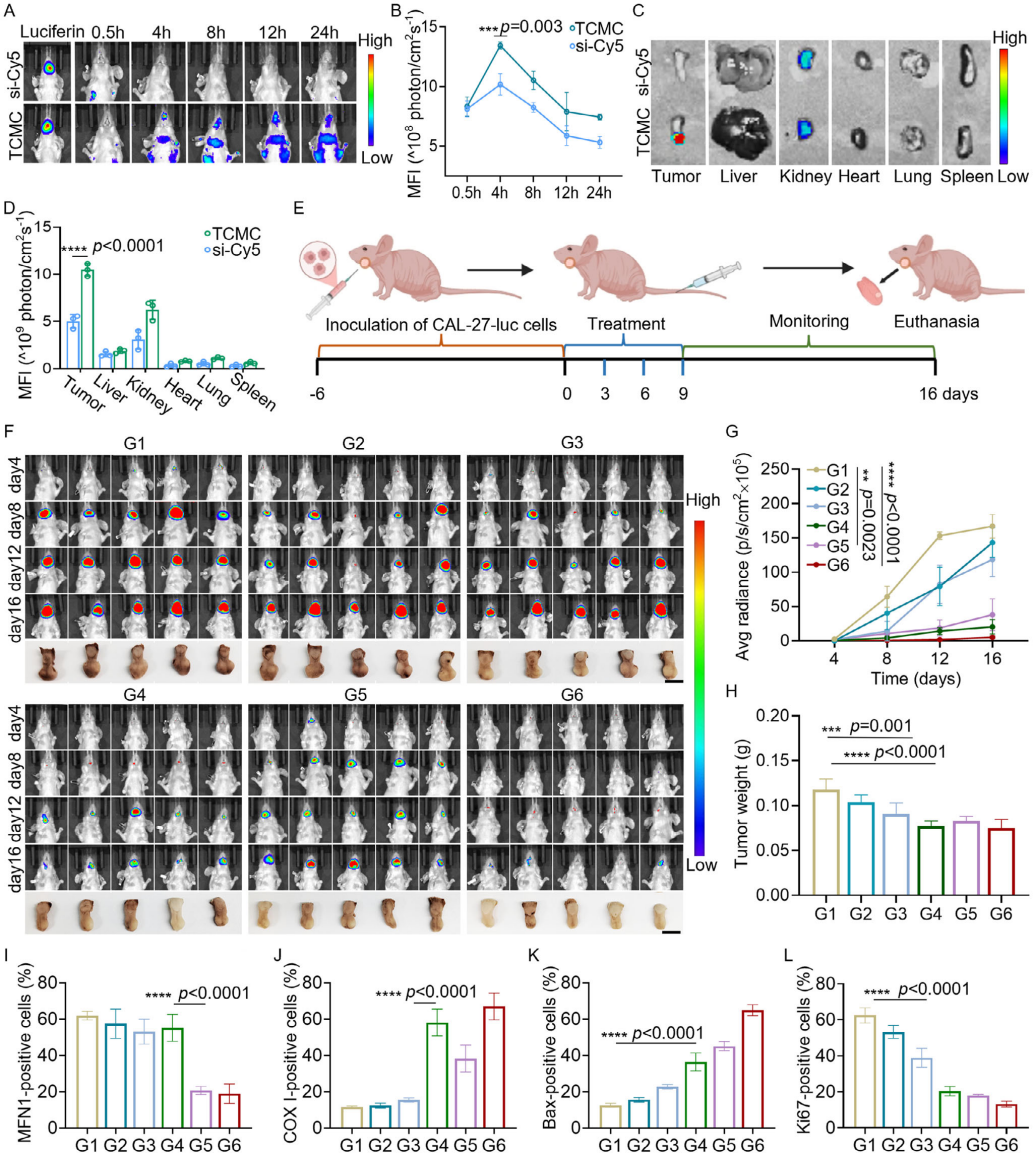
Biodistribution and antitumor effect of TCMH in the OSCC CDX model. (A) In vivo FL imaging of TCMH at different timepoints. (B) Quantification of the corresponding FL intensity of tumor sites. (C) Ex vivo FL imaging of organs at 4 h postinjection. (D) Quantification of the corresponding FL intensity of major organs at 4 h postinjection. (E) Schematic illustration of the establishment of OSCC CDX model and treatment process. G1: PBS, G2: TA, G3: TC, G4: TCH, G5: TCM, G6: TCMH. (F) Representative tongue images and bioluminescence images of the tumors measured on day 4, 8, 12, and 16. The scale bar was 100 mm. (G) Quantitative FL intensity analysis of the tumors measured on day 4, 8, 12, and 16. (H) Tumor weight of tumors after 16 days of treatment. (I–L) Immunohistochemical analysis of MFN1, COX I, Bax, and Ki67‐positove cells in tumor tissue sections. The data were presented as mean ± SD (*n* = 5). Statistical significance was calculated via one‐way ANOVA with Tukey's test: *p < 0.05, **p < 0.01, ***p < 0.001, and ****p < 0.0001.

To evaluate tumor accumulation of TCMH, an orthotopic OSCC model was first established by subcutaneous injection of luciferase‐expressing Cal‐27 cells (Cal‐27‐luc) into tongues of nude mice. 7 days post‐injection, luciferase activity confirmed successful tumor implantation. FL imaging results indicated that TCMC enriched in tumor region rapidly and reached a plateau at 4 h postinjection. (Figures 6A,B and S22). After 24 h, major organs and tumors were harvested for ex vivo imaging. At 4 h post‐administration, tumors displayed the highest FL intensity of TCMC compared with that in other organs. In contrast to TCMC group, the tumoral FL signal in free si‐Cy5 group was found to be negligible (Figure 6C,D).

Inspired by the excellent tumor targeting ability of TCMH, we further evaluate their therapeutic efficacy in the in situ luciferase‐overexpressing OSCC CDX model (Figure 6E). At day 9, the model was successfully established. Mice were randomly grouped and intravenously administrated with various formulations, including PBS (G1), TA (G2), TC (G3), TCH (G4), TCM (G5), TCMH (G6) every 3 days (Figures 6E and S23). Bioluminescence images and quantitative analysis reflected the antitumor effects. Compared with PBS and TA groups, TC led to only a certain degree of tumor suppression owing to the released Ca^2+^ within tumor sites, activating Ca^2+^ interference. In contrast, cooperation of siMFN1 in G5 accelerated the tumor inhibition process, as evidenced by a low luminescence signal. Thanks to H_2_S and Ca^2+^ overload‐mediated efficient ROS production, G4 elicited a more obvious antitumor effect than G1, G2, G3, G5 (Figures 6F,G and S23). The most potent tumor control was observed in G6 group, which might be attribute to the synergy of mitochondrial fusion and mitochondrial function regulation. In nude mice bearing orthotopic tongue OSCC tumors, control animals displayed persistent tumor proliferation. Growing tumors occupied the oral cavity to hinder food intake, leading to progressive body weight loss at the late experimental stage; this phenomenon constituted an inherent limitation of the model. In this study, G1 and G2 were controls without effective drug intervention, whereby their tumors grew rapidly and nearly occupied the entire oral cavity of mice, with impaired food intake being the most prominent complication. Accordingly, mice in these two groups exhibited markedly greater body weight loss relative to treated Groups 3–6. The experiment was immediately terminated when transient weight loss exceeding 20% was observed in a few mice from the G1 and G2 groups on Day 16 (Table S3 and Figure S24). Notably, G6 displayed the slowest weight loss rate, which correlated with the smallest tumor weights, suggesting reduced tongue tumor size alleviated feeding impairment (Figure 6H). The H&E staining results also revealed the smallest tumor lesions in G6 (Figure S25). Immunohistochemical analysis results revealed remarkable lowest percentage of MFN1‐positive cells (18.90 ± 4.33%) in TCMH ‐treated mice (Figures 6I and S26). In contrast, COX I proteins were much upregulated in G4‐G6, indicating increased ROS‐mediated tumor cytotoxicity (Figures 6J and S26). TCMH had highest level of pro‐apoptotic Bax (64.90 ± 2.44%) and lowest percentage of cell proliferation marker Ki‐67‐positive cells (13.31 ± 1.31%) (Figures 6K,L and S26). These findings collectively verified the super antitumor effect of TCMH. Peripheral blood biochemical analyses (ALT, AST, TP, ALP, BUN, CR) revealed no abnormalities in all groups (Figure S27). H&E staining of major organs showed the absence of histopathological lesions in TCMH group, further confirming their biocompatibility (Figure S28).

### 2.7 Antitumor Performance in the PDX Model

**FIGURE 7:**
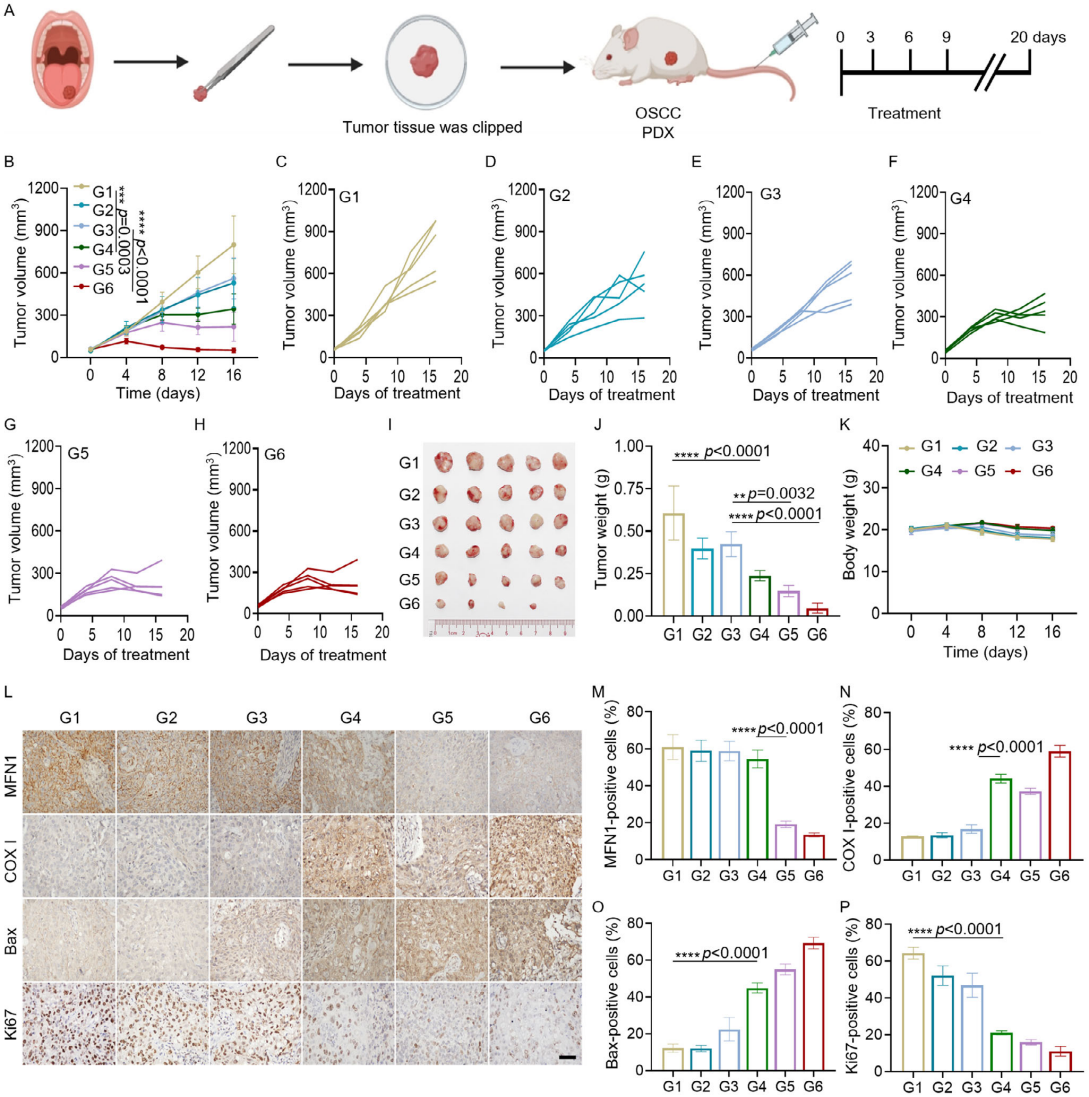
Antitumor effect in the OSCC PDX model. (A) Schematic illustration of the establishment of OSCC PDX model and treatment process. G1: PBS, G2: TA, G3: TC, G4: TCH, G5: TCM, G6: TCMH. (B) Tumor growth profiles of tumors after different treatments. (C–H) Tumor volume curves from G1 to G6. (I) Representative photographs of PDX tumors after 16 days of treatment. (J) Representative weights of PDX tumors after 16 days of treatment. (K) Body weight changes during treatment. (L) Immunohistochemical images of MFN1, COX I, Bax, and Ki67‐positive cells in tumor tissue sections. The scale bar was 10 µm. (M–P) The corresponding quantitative analysis of MFN1, COX I, Bax, and Ki67‐positive cells in tumor tissue sections. The data were presented as mean ± SD (*n* = 5). Statistical significance was calculated via one‐way ANOVA with Tukey's test: *p < 0.05, **p < 0.01, ***p < 0.001, and ****p < 0.0001.

To further validate the clinical translatability of TCMH, we accessed the antitumor performance in a PDX model. The MFN1‐positive OSCC patient tumor tissue samples were collected for PDX model construction. The treatment process was similar to the above CDX model (Figure 7A). Mice were randomly grouped and intravenously administrated with various formulations, including PBS (G1), TA (G2), TC (G3), TCH (G4), TCM (G5), TCMH (G6) every 3 days. An inapparent decrease of tumor volume observed in G2 and G3 groups indicated their weak tumor inhibition effect (Figure 7B–E). TCM exhibited better tumor growth suppressive efficacy than TCH, indicating the tumors in PDX model exhibited greater sensitivity to the therapeutic strategy of MFN1 silencing in combination with Ca^2^^+^ overload. (Figure 7B,F,G). Even after 20 days treatment, significant lowest tumor volume was observed in G6 (Figure 7H), and the presence of the smallest tumors in this group further confirmed their tumor inhibition effect (Figure 7I). The corresponding lowest tumor weight and H&E staining of tumor tissues re‐demonstrated its super therapeutic performance (Figure 7J). Slight body weight loss was observed in G1‐G3. (Figure 7K, Table S4). This was mainly due to the inherent heterogeneity of OSCC that impaired the general health status of mice and consequently affected their body weight changes. TCMH potently inhibited tumor proliferation and induced tumor apoptosis, as evidenced by the lowest level of MFN1 and Ki67 and the highest levels of Bax and COX I in tumor tissues (Figure 7L–P). Blood biochemical assays and H&E staining of major tissues revealed the excellent biocompatibility and systemic safety of TCMH (Figures S29 and S30). Collectively, TCMH possessed enhanced antitumor potency in both CDX and PDX models by triggering tumor‐specific mitochondria apoptosis.

## 3 Conclusion

In recent years, tumor intervention strategies targeting mitochondrial Ca^2+^ overload have become a research hotspot in the field of anti‐tumor therapy. Through multitherapeutic modes combination, Ca^2+^ nanomodulators can elevate mitochondrial Ca^2+^ concentration by stimulating Ca^2+^ release from the endoplasmic reticulum to the cytoplasm, enhancing Ca^2+^ influx and suppression of cell membrane Ca^2+^ efflux [51, 52, 53, 54, 55]. However, mitochondria initiate Ca^2^^+^ metabolic autoregulation via Ca^2+^ channel proteins. MCU modulation‐mediated Ca^2+^ overload could overcome this challenge. Current studies primarily employ small molecule inhibitors (such as DS16570511, Ru265) [8, 56, 57] mitoxantrone, berberine, fluoride, and other chemical drugs to directly modulate MCU function [9, 13, 15, 16, 23]. However, these interventions are low targeting specificity, which may disrupt Ca^2+^ homeostasis in normal tissues and result in insufficient biological safety. Researchers also have engineered nanomodulators to trigger endogenous Ca^2+^ flux through a multistage cascade via synergistic miRNA ‐responsive MCU modulation and TRPA1 channels activation [58]. But this strategy does not trigger Ca^2+^ overload merely by regulating MCU alone.

In our study, based on clinical samples analysis of OSCC, we confirmed that MFN1 was abnormally highly expressed in tumor tissues and was a key pathogenic target for OSCC therapy (Figure 1). Notably, MFN1‐mediated mitochondrial fusion is closely to mito‐Ca^2^^+^ homeostasis regulation and activate MCU for mito‐Ca^2+^ surge [22, 23, 24, 59]. Furthermore, H_2_S can trigger specific mito‐Ca^2+^ surge by opening MCU for cytosolic Ca^2+^ influx via downregulation of MCU1 [25]. Thus, different from current strategies on MCU modulation, we proposed a direct MCU‐mediated mito‐Ca^2^^+^ metabolic autonomy regulation strategy mainly based on synergistic effect of mitochondrial fusion suppression and H_2_S. We developed a metal‐phenolic nanocluster (TCMH), in which siMFN1, H_2_S donor, and Ca^2+^ are coordinated with polyphenols. This nanocluster boasted superior attributes (e.g., strong metal chelation ability, rapid coordination process, and pH responsiveness) of rich phenolic groups (Figure 2). TCMH‐mediated MFN1 deficiency reshaped the mitochondrial fission and fusion homeostasis to relieve the inherent inhibition of MCU, priming potent Ca^2^^+^ influx in mitochondria and subsequent mitochondrial depolarization (Figures 4, S9, S10, and S17). Meanwhile, H_2_S released from TCMH also strengthened this MCU activation pathway, thereby triggering rapid surges in mito‐Ca^2+^ distribution and Ca^2+^ overload. (Figures 4 and 5). We had verified the synergistic MCU modulation effect of MFN1 suppression and H_2_S (Figure S18). siMFN1‐mediated mitochondria fusion inhibition and H_2_S activated MCU channels to facilitate endogenous Ca^2+^ influx into mitochondria. Additionally, exogenous Ca^2^^+^ introduction in this nanosystem significantly enhanced mito‐Ca^2+^ overload (Figure S18). This nanocluster further boosted ROS production, forming a self‐amplifying ROS‐Ca^2+^ feedback loop that robustly activated the mitochondrial apoptotic pathway and ultimately driven cell apoptosis (Figure 5). We also verified the application potential of this strategy in various tumor types, providing a solid clinical basis and broader application prospects (Figures S13 and S14). Eventually, in vivo antitumor results further verified TCMH's strong tumor suppression performance, as well as biocompatibility and biosafety (Figures 6 and 7).

In summary, this promising MCU‐mediated mito‐Ca^2+^ metabolic autonomy modulation strategy has superior therapeutics and provides a paradigm for mitochondria‐targeted tumor Ca^2+^ interference therapy.

## 4 Experimental Section

### 4.1 Statistical Analysis

All measurements were conducted on three or more independent replications from different experiments. Statistical analysis was performed using Graphpad Prism 8. Data are presented as mean ± SD. Spearman correlation analyses and an unpaired *t*‐test were used to examine the correlation MFN1 expression in OSCC patients. One‐way analysis of variance (ANOVA) followed by Tukey's test was used for comparison between two groups or among multiple groups, respectively. Probability *p* < 0.05 was considered to be statistically significant: *p < 0.05, **p < 0.01, ***p < 0.001, and ****p < 0.0001.

### 4.2 Ethical Approval

This study was conducted in accordance with international guidelines and the ethical standards outlined in the Declaration of Helsinki. The OSCC tumors tissues samples from human were collected for tumor markers analysis and PDX model construction. Informed consent was obtained from all participants, in accordance with ethical guidelines. The protocols were approved by the Ethics Committee of Sun Yat‐sen Memorial Hospital (Number: SYSKY‐2025‐487‐03). All animal experiments were performed in compliance with institutional guidelines following protocol approval by the Ethics Committee of the Animal Center, Medical Research Center, Sun Yat‐sen Memorial Hospital (Approval No.: AP20250085). All animal experiments were immediately terminated if any animal presented transient body weight loss exceeding 20%, in accordance with the ARRIVE 2.0 guidelines and national Ministry of Health regulations governing the housing and welfare of laboratory animals.

Detailed materials and methods can be found in the Supporting Information.

## Author Contributions

**Ronglong Chen**: methodology, investigation, conceptualization, writing – original draft, data curation. **Chunxue Song**: visualization, formal analysis, software. **Juan Liu**: conceptualization, methodology, formal analysis. **Shucong Yao**: conceptualization, investigation, validation. **Xijun Lin**: investigation, methodology. **Zixian Huang**: writing – review and editing, supervision, funding acquisition. **Yuepeng Wang**: methodology, investigation, visualization. **Li Lin**: conceptualization, resources, investigation. **Fei Wu**: investigation, validation, data curation. **Yingjie Deng**: investigation, conceptualization. **Jia Huang**: methodology, investigation, validation, formal analysis. **Chao Zhang**: supervision, funding acquisition, resources. **Lisi Xie**: funding acquisition, writing – review and editing, supervision, project administration. **Zhiquan Huang**: funding acquisition, supervision.

## Conflicts of Interest

The authors declare no conflicts of interest.

## Supporting information




**Supporting File**: advs76243‐sup‐0001‐SuppMat.docx.

## Data Availability

The data that support the findings of this study are available from the corresponding author upon reasonable request.
