# Supplementary material for: A Metal‐Phenolic Nanocluster Orchestrates Mito‐Ca2+ Metabolic Autonomy for Tumor Ca2+ Interference Therapy
Source: Adv Sci (Weinh). 2026 Jun 22;13(52):e76243. doi: 10.1002/advs.76243 (PMC13336888; doi:10.1002/advs.76243)
Supplement: Supplementary file 1 [file ADVS-13-e76243-s001.docx]

Copyright WILEY-VCH Verlag GmbH & Co. KGaA, 69469 Weinheim, Germany, 2020.

Supporting Information

**A Metal-Phenolic Nanocluster Orchestrates** **Mito-Ca^2+^** **Metabolic Autonomy for** **Tumor Ca^2+^ Interference Therapy**

Ronglong Chen^†^, Jia Huang^†^, Yuepeng Wang, Shucong Yao, Fei Wu, Juan Liu, Chunxue Song, Li Lin, Fei Wu, Xijun Lin, Yingjie Deng, Chao Zhang*, Zhiquan Huang*, Zixian Huang*, Lisi Xie*

R. Chen, J. Liu, C. Song, L. Lin, X. Lin, Y. Deng and Prof. L. Xie

Guangdong Provincial Key Laboratory of Malignant Tumor Epigenetics and Gene Regulation, Medical Research Center, Sun Yat-Sen Memorial Hospital, Sun Yat-Sen University, Guangzhou 510120, China *Email: [xiels6@mail.sysu.edu.cn](mailto:xiels6@mail.sysu.edu.cn) (L. Xie)

Prof. J. Huang

Reproductive Medicine Center, The First Affiliated Hospital, Sun Yat-sen University, Guangzhou 510080, China

S. Yao

Department of Oral and Maxillofacial Surgery, Nanfang Hospital, Southern Medical University, Guangzhou, China

Dr Fei Wu

Department of Musculoskeletal Oncology, The First Affiliated Hospital, Sun Yat-sen University, Guangzhou 510080, China

Prof. Z. Huang

Department of Oral and Maxillofacial Surgery, Sun Yat-sen Memorial Hospital, Sun Yat-sen University, Guangzhou 510120, PR China

*E-mail: [hzhquan@mail.sysu.edu.cn](mailto:hzhquan@mail.sysu.edu.cn) (Z. Huang)

Y. Wang and Dr Z. Huang

Department of Oral and Maxillofacial Surgery, Sun Yat-sen Memorial Hospital, Sun Yat-sen University, Guangzhou 510120, PR China; Nanhai Translational Innovation Center of Precision Immunology, Sun Yat-Sen Memorial Hospital, Foshan 528200, PR China

*E-mail: [huangzx66@mail.sysu.edu.cn](mailto:huangzx66@mail.sysu.edu.cn) (Z. Huang)

Prof. C. Zhang

Beijing Institute of Basic Medical Sciences, Beijing 100850, China

*E-mail: [zhangchao0103@163.com](mailto:zhangchao0103@163.com) (C. Zhang)

†R. Chen and J. Huang contributed equally to this work.

*Materials*: DMEM (Dulbecco’s Modified Eagle Medium) were purchased from Gibco Life Sciences. Fetal bovine serum (FBS) was obtained from Shanghai Dartshill Biotechnology Co., Ltd. Trypsin-EDTA (0.25%) was purchased from MedChemExpress. Cell Counting Kit-8 (CCK-8), and d-fluorescein (potassium salt) were obtained from APExBio Co., Ltd. Rhod-2 AM (C3276) were obtained from APExBio Co., Ltd. Commercial transfection reagent Lipo 3000 was purchased from Thermo Fisher Scientific. siMFN1-cy5 (si-Cy5) and siRNA-MFN1 (siMFN1) were purchased from Suzhou Hongxun Technology Co., LTD. DAPI Solution was purchased from Beijing Sora Biotechnology Co., LTD. Mitochondrial membrane potential assay kit was purchased from Shanghai Tianyu Biotechnology Co., LTD. Methanol, anhydrous ethanol, tannic acid, calcium chloride, 4% paraformaldehyde were purchased from Shanghai Aladdin Biochemical Technology Co., Ltd. DCFH-DA was purchased from MedChem Express. RIPA Lysis Buffer, BCA Protein Assay Kit, 1 mM PMSF, 1× protease inhibitor cocktail were purchased from Jiangsu Kangwei Century Biotechnology Co., Ltd. Skim milk was purchased from Biosharp Life Sciences. The enhanced ATP assay kit and Fluo-4, AM were purchased from Beyotime Biotechnology. Anti-MFN1 antibody (A5441, 1:3000 dilution for western blot, 1:200 dilution for immunohistochemistry), Anti-Bax antibody (50599, 1:4000 dilution for western blot, 1:400 dilution for immunohistochemistry), Anti-caspase-3 antibody (9505, 1:5000 dilution for western blot), Anti-MCU antibody (26312, 1:2000 dilution for western blot), Anti-COX I antibody (13393, 1:2000 dilution for western blot, 1:400 dilution for immunohistochemistry), Anti-β-actin antibody (5174, 1:10000 dilution for western blot), HRP goat anti-rabbit IgG H&L (RGAR001, 1:5000 dilution for western blot) was purchased from Wuhan Sanying Biotechnology. Anti-Bcl-2 antibody (Ab182858, 1:1000 dilution for western blot) was purchased from Abcam. Anti-Ki-67 antibody (GB111141, 1:1000 dilution for immunohistochemistry) was purchased from Servicebio Technology Co., Ltd. Mouse Cytochrome C (Cyt C) ELISA Kit (EM0977) was purchased from FineTest.

*Characterization:* ^1^H NMR spectra were recorded on NMR (Bruker AVIII 500HD) spectrometers, using tetramethylsilane as an internal standard, the deuterated dimethyl sulfoxide (DMSO-*d6*) were used as the solvents.Transmission electron microscopy (TEM) images were acquired on a Hitachi HT7800 microscope operated at an acceleration voltage of 120 kV. Size distribution and stability of nanoparticles were determined by dynamic light scattering (Zetasizer Nano, Malvern) at 25 °C. Energy dispersive X-ray spectroscopy (EDS) and elemental mapping analysis were measured by FEI Tecnai G2 F30 S-Twin TEM/STEM microscope at 300 kV. The concentration of Ca were detected by the iCAP^TM^ Q inductively coupled plasma mass spectrometry (ICP-MS) (Agilent 7800 instrument). The UV-Visible-NIR absorption spectra were acquired via UV-Visible Spectrophotometer Evolution 201. Fluorescence spectra were obtained by a fluorescence spectrometer (Fluoresecence Spectrophotometer F-4700, HITACHI). Confocal microscopy images were acquired on a Olympus FV3000 confocal microscope.The fluorescence images of cells were collected by fluorescent microscopy (Olympus IX73).

*Synthesis of PEG modified polyphenols (PEG-DA)* The PEG modified polyphenols were sysnthesized following a published protocol^[1]^. 8-arm PEG-SG (1 g) and dopamine hydrochloride (380 mg) were dissoloved in DMF solution (10 mL) under argon protection for 2 h. Then TEA (200 μL) was added into the above solution under argon protection overnight. The obtained product was purified by dialysis for 3 days to remove the free components, and then lyophillizated.

*Synthesis of methoxyl polyethylene glycol-2-poly[(2-methacrylamidoethyl) carbamodithioate]-2-methylpropanoate (PEG-DTC).* The synthesis of PEG-DTC was according to our published method described in our previous study^[2]^. methoxyl polyethylene glycol-2-poly[N-(2-aminoethyl) methacrylamide]-2-methylpropanoate (PEG-EDA) (100 mg) and NaOH (2 mmol, 80 mg) was mixed and dissolved by methanol in an ice-water bath for 2 h. carbon disulfide (2 mmol, 152 mg) was added to react for another 4 h at room temperature. The solution was then filtrated and concentrated. The concentrated crude product was purified by precipitation in diethyl ether to obtain PEG-DTC with the yield of 130 mg. Mw = 6032, PDI = 1.51 (5 DTC moieties in each conjugated polymer). 1H NMR (DMSO-*d6*, Bruker AVIII 500HD, 298 K, ppm) was shown in Figure S4.

*Preparation of TCMH.* Firstly, TA (53.5 μL, 15 mg mL^-1^), siMFN1/si-Cy5 (10 pmol), PEG polyphenols (17.7 μL, 15 mg mL^-1^) and CaCl_2_ (100 μL, 1mg mL^-1^) were mixed in 3 ml milli-Q water and stirred for 2 h at 1200 rpm. Then, the PEG-DTC (60 μL, 4 mg mL^-1^) was added in the mixture for further stirring of 30 min. Afterward, the TCMH NPs were purified by ultrafiltration for 3 times (2500 rpm, 10 min). Finally, the final product was obtained and stored in 1 mL milli-Q water in 4 °C. The encapsulation efficiency of TA and si-Cy5 was calculated by characteristic UV-Vis absorbance value of TA at 270 nm and fluorescence intensity of si-Cy5 respectively. The preparation of TC, TCM and TCH was refer to the above protocol.

*siRNA gel electrophoresis*. Different proportions of TCMH NPs with the same amount of siMFN1 was mixed with 5 μL of DNA Loading Buffer. The resulting mixtures were loaded onto a 1.0% agarose gel and subjected to electrophoresis in TAE buffer at a constant voltage of 120 V for 20 minutes. Finally, the gel was imaged using a nucleic acid gel imaging system (Champgel 6000).

*pH responsive disassembly behaviors of TCMH.* To determine pH responsive disassembly, TCMH were treated at different pH (5.0 and 7.4) solutions. The characteristic absorption at 270 nm of released TA (1 mg mL^−1^) was measured by ultraviolet spectrophotometer and the release ratio was calculated.

*Detect H_2_S with the Elman reagent.* The linear relationship between the absorption spectra of 5,5'-dithiobis (2-nitrophenylacetic acid) and GSH was determined by titration. In PBS with pH values of 5.5, 6.6, 7.4, and 9.0, pH-responsive release of H_2_S from TCMH (1.1 μM) was also detected using 5,5'-dithiobis (2-nitrophenylacetic acid). The optical density (OD) at 412 nm was measured using a BioTek H1 multimode microplate reader.

*Intracellular H_2_S Detection.* The H_2_S in the TCMH-treated cells was stained with the commercial WSP-5 fluorescent probe. Cal-27 cells were seeded in 12-well plates at a density of 80,000 cells per well. The adherent cells were treated with WSP-5 (50 μM, 1 mL) at 37°C for 30 minutes. The stained cells were analyzed using CLSM.

*Cell culture and animals*. The human HNSCC cell lines (CAL-27), and HEK293t were obtained from the American Type Culture Collection (ATCC). All cell lines were routinely cultured in DMEM supplemented with 10% FBS, penicillin (100 U mL^-1^), and streptomycin (100 μg mL-1) in a 37°C humidified incubator containing 5% CO_2_. All the cell lines were validated by short tandem repeat (STR) profiling analysis and were free of mycoplasma contamination. To construct the Cal-27 cell line with stable mitochondrial expression (Cal-27-mito-GFP), mitochondrial-targeted fluorescent plasmids were transfected into parental Cal-27 cells using Lipofectamine 293^TM^. Transfected cells were selected with 2.5 μg mL^-1^ puromycin for 2-3 weeks, and single-cell clones with uniform mitochondrial fluorescence (confirmed by confocal microscopy) were picked and expanded. The stable cell line was cultured in DMEM supplemented with 10% FBS and 1% penicillin-streptomycin at 37 °C with 5% CO₂, and maintained with 2.0 μg mL^-1^ puromycin to preserve plasmid stability. The cells were cultured in 37°C incubator with humidified atmosphere of 5% CO_2_. BALB/c-Nude female mice (4-6 weeks, 18-20 g) were provided by GemPharmatech Co., Ltd and raised in a specific pathogen free environment.

*Cellular uptake.* The cell uptake behavior of TCMH was evaluated using CLSM and flow cytometry analysis. Cal-27 cells were inoculated in 12-well plates at a density of 20,000 cells per well and incubated for 24 hours. The cells were then incubated with TCM containing si-Cy5 for 0, 4, 6, 8, 12 hours. For CLSM imaging, the cells were washed 3 times with PBS, fixed with 4% paraformaldehyde and DAPI and the images were obtained by CLSM. For flow cytometry analysis, cells are washed with PBS, digested by trypsin, collected by centrifugation, and then analyzed by flow cytometry.

*The evaluation of cellular MFN1 expression level.* Cal-27 cells were inoculated in 6-well plates with a density of 1 × 10^5^ per well for 24 hours. Thereafter, the medium was replaced with fresh medium with siMFN1 loaded TCMH (siMFN1: 50 nM) NPs. After incubation for 48 h, the cells were collected for qPCR and western blotting analysis of MFN1 expression.

*Mitochondrial fusion inhibition*. In order to investigate mitochondrial fusion inhibition, Cal-27 -mito-GFP cells were inoculated on 12-well plates at first. The cells were then transfected with different formulars including TA, TC, TCM, TCH and TCMH (siMFN1: 50 pmol mL^-1^, TA: 100 μg mL^-1^, Ca^2+^: 30 μg mL^-1^, PEG-DTC: 243.9 μg mL^-1^) and treated for 24 hours. After 24 hours, for CLSM imaging, cells were washed 3 times with cold PBS, fixed with 4% PFA and stained by DAPI, and then the images of mitochondrial morphology and cell morphology were obtained by CLSM.

*ROS detection*. The cells were seeded in a 6-well plate at a density of 1×10^5^ per well and incubated overnight. Then the cells were treated with TA, TC, TCH, TCM, TCMH respectively (siMFN1: 50 pmol mL^-1^, TA: 100 μg mL^-1^, Ca^2+^: 30 μg mL^-1^, PEG-DTC: 243.9 μg mL^-1^) for 24 hours. After that, the cells were washed by PBS and incubated with 10 μM of DCFH-DA at 37 °C for 20 min. Then, the cells were washed 3 times with PBS, the cells were fixed with 4% PFA and stained with DAPI, following by collecting images via a fluorescence microscope. The quantification of ROS production was measured using a CytoFLEX flow cytometer. Flow Jo software was used for data analysis.

*Intracellular Ca^2+^ detection*. Cellular Ca^2+^ levels were measured using a calcium ion probe Fluo-4, AM. The cells were inoculated in a 6-well plate at a density of 1×10^5^ per/well and incubated overnight. Subsequently, the cells were treated with TA, TC, TCH, TCM, TCMH respectively for 24 hours (siMFN1: 50 pmol mL^-1^, TA: 100 μg mL^-1^, Ca^2+^: 30 μg mL^-1^, PEG-DTC: 243.9 μg mL^-1^). Next, the cells were washed with PBS 3 times, and stained with 4 μM Fluo-4 for 20 min. Then, images were acquired by using a fluorescence microscope. The quantification of Ca^2+^ production was measured using a CytoFLEX flow cytometer. Flow Jo software was used for data analysis.

*Mitochondrial Ca^2+^ detection.* The detection of intramitochondrial calcium ion concentration was performed using a Rhod-2 AM fluorescence probe. The cells were inoculated in a 6-well plate at a density of 1×10^5^ per/well and incubated overnight. Subsequently, the cells were treated with TA, TC, TCH, TCM, TCMH respectively for 24 hours (siMFN1: 50 pmol mL^-1^, TA: 100 μg mL^-1^, Ca^2+^: 30 μg mL^-1^, PEG-DTC: 243.9 μg mL^-1^). Next, the cells were washed with PBS 3 times, and then loaded with a working solution containing 5 μM Rhod-2 AM for 30 minutes in a 37°C cell incubator in the dark. Subsequently, the loading solution was discarded, and the cells were washed three times with pre-warmed PBS to remove residual probes. Then, the medium was replaced with complete medium, and the cells were further incubated in the dark for 20 minutes to ensure complete de-esterification of the probe and its localization in mitochondria. Then, images were acquired by using a fluorescence microscope. The quantification of Rhod-2 production was measured using a CytoFLEX flow cytometer. Flow Jo software was used for data analysis.

*Cell Cytotoxicity and proliferation*. The cells were cultured in 96-well plates at a density of 5×10^3^ per well and incubated overnight. For cell cytotoxicity evaluation, the cells were treated with PBS, TA, TC, TCH, TCM, TCMH respectively for 24 hours. For cell proliferation assay, the cells were treated with TA, TC, TCH, TCM, TCMH respectively for 1, 2, 3, 4, 5 days (siMFN1: 50 pmol mL^-1^, TA: 100 μg mL^-1^, Ca^2+^: 30 μg mL^-1^, PEG-DTC: 243.9 μg mL^-1^). After that, the cells were incubated with 100 μL serum-free medium with CCK-8 solution for 1 h. Then the absorbance was measured at 450 nm (OD value) with an enzyme-labeled instrument (BioTek H1). Cell viability is calculated as following fomula: Cell viability (%) = (OD _sample_-OD _blank_/ (OD _control_-OD _blank_) ×100%.

*Cell colony.* The cells were cultured in a 6-well plate at a density of 1×10^3^ per well and incubated for 24 hours. the cells were treated with PBS, TA, TC, TCH, TCM, TCMH respectively for 7-10 days (siMFN1: 50 pmol mL^-1^, TA: 100 μg mL^-1^, Ca^2+^: 30 μg mL^-1^, PEG-DTC: 243.9 μg mL^-1^). After 7 days of culture, the cells were washed with PBS for 3 times, fixed with 4% PFA for 15 minutes, stained with 0.1% crystal violet solution for 10 minutes, rinsed slowly with H_2_O and waited for the moisture to dry. The photographs were collected. The number of cells was analyzed with ImageJ software.

*Cell Apoptosis.* The cells were cultured in a 6-well plate at a density of 1×10^3^ per well and incubated for 24 h. The cells were treated with PBS, TA, TC, TCH, TCM, TCMH respectively for 36 h (siMFN1: 50 pmol mL^-1^, TA: 100 μg mL^-1^, Ca^2+^: 30 μg mL^-1^, PEG-DTC: 243.9 μg mL^-1^). After incubation, the cell was then collected and washed according to the kit manufacturer's instructions for subsequent experiments. The cells were suspended in the binding buffer with 10 μL Annexin V-FITC and 5 μL PI solutions respectively, and incubated for 15 min. The apoptosis rate was detected by flow cytometry and the data were analyzed by Flow Jo software version 10.

*Mitochondrial damage*. The JC-1 Mitochondrial membrane Potential Measurement kit was used to measure mitochondrial membrane potential (ΔΨm). The cells were cultured in a 6-well plate at a density of 2×10^5^ cells per well and incubated for 24 hours. The cells were treated with PBS, TA, TC, TCH, TCM, TCMH respectively for 24 h (siMFN1: 50 pmol mL^-1^, TA: 100 μg mL^-1^, Ca^2+^: 30 μg mL^-1^, PEG-DTC: 243.9 μg mL^-1^). Then, the cells (2×10^5^) were re-suspended in 0.5 mL JC-1 probe working solution and incubated at 37 °C for 20 minutes. The cells were further analyzed by CLSM and flow cytometry.

*Mitochondrial ATP content detection*. An enhanced ATP assay kit based on luciferase detection was used to determine ATP levels. The cells were cultured in a 6-well plate at a density of 2×10^5^ cells per well and incubated for 24 hours. The cells were treated with PBS, TA, TC, TCH, TCM, TCMH respectively for 24 h (siMFN1: 50 pmol mL^-1^, TA: 100 μg mL^-1^, Ca^2+^: 30 μg mL^-1^, PEG-DTC: 243.9 μg mL^-1^). The cells were collected and added with 100 μL ATP detection working solution. The luminescence was detected by multifunctional enzyme marker. Intracellular ATP levels were calibrated by protein concentration and ATP standard curve of each group.

*RNA extraction and real-time quantitative RT-PCR*. Total RNA was extracted using an RNA extraction kit according to the instructions and then reverse-transcribed into cDNA using PrimeScript TM RT Premix (Takara, Japan) on the ABI 9700 RealTime PCR system (ABI, USA). The newly synthesized cDNA was then used as a template for detecting the desired genes. Specifically, 1 μL cDNA was mixed with TB Green®Premix Ex TaqTM II (Takara, Japan) to react 20 μL. Three reactions were performed using the above primers. The reaction conditions were as follows: 94°C 2min, 94°C 20s, 58°C 20s and 72°C 2s 40 cycles. Relative mRNA expression was detected by Roche LightCycler 480 II real-time fluorescent quantitative PCR assay (Roche, USA). The primers of Gapdh are 5 '-AgATCCCTCCAAAATCAAgTgg-3' and 5 '-ggCAgAgATgATgACCCTTTT-3', The primers of MFN1 are 5 '-CCAGGTACAGATGTCACCACAG-3' and 5 '-TTGGAGAGCCGCTCATTCACCT-3'.

*Western blotting analysis*. The cells were treated with PBS, TA, TC, TCH, TCM, TCMH respectively for 24 h (siMFN1: 50 pmol mL^-1^, TA: 100 μg mL^-1^, Ca^2+^: 60 μg mL^-1^, PEG-DTC: 243.9 μg mL^-1^). All lysates from each group were collected to obtain protein samples for western blotting analysis. BCA protein detection kit was used to quantify the concentration of total protein. The 20 μg protein sample was isolated on 10% and 12.5% SDS-PAGE gel and electrophoretic in TRIS-Glycine buffer at constant voltage 80/120 V. The protein sample was transferred from the gel at a constant voltage of 200 mA to the PVDF membrane for 70 min. The membranes were sealed with 5% skimmed milk for 1 h at room temperature and then incubated with primary antibody at 4 °C overnight. The primary antibodies dilutions were as follows: β-actin (Proteintech, 5174, 1:50000), MFN1 (Proteintech, A5441, 1:2000), Bax (Proteintech, 50599-2, 1: 4000), Bcl-2 (Abcam, Ab182858, 1:1000), caspase-3 (Proteintech, 9505, 1: 5000), MCU (Proteintech, 26312-1-AP, 1:2000) and COX I (Proteintech, 13393-1-AP, 1:2000). Then the membrane was washed by TBST 3 times, and incubated with the corresponding secondary antibody for 1 hour at room temperature, away from light. Then, Protein bands were developed with ECL developer and reacted with ECL detection reagent. The bands were observed with an image imager (MiniChemi 910).

*Cytochrome C detection.* Cytochrome C-based ELISA kit was used to detect the content of cytochrome C. The cells were incubated in 6-well plates at a density of 2×10^5^ cells per well for 24 hours. After the cells were treated with PBS, TA, TC, TCH, TCM, TCMH respectively for 24 h (siMFN1: 50 pmol mL^-1^, TA: 100 μg mL^-1^, Ca^2+^: 30 μg mL^-1^, PEG-DTC: 243.9 μg mL^-1^), the cells were lysed and collected for ELISA detection. Then, the absorbance of samples was measured by the enzyme-labeled instrument. Finally, the content of cytochrome C was quantified by the standard curve. Mouse Cytochrome C (Cyt C) ELISA Kit (EM0977) was purchased from FineTest.

*Biodistribution*. Cal 27-stable overexpressed luciferase tumor-bearing mice were used for in vivo fluorescence imaging. The mice were injected intravenously by TCMH containing Cy5-siRNA at a dose of 1 nmol siRNA per mouse. The mice intravenously injected with Cy5-siRNA was as the control. The Mice were imaged at different timepoints using the IVIS system (Cri Inc.). Organs and tumors are then collected and imaged.

*In vivo antitumor performance evaluation in an* *in situ CDX model*. To investigate the antitumor effect of TCMH in vivo, *in situ CDX model was* conducted using Cal-27 luciferase and MFN1 stable overexpressed cell lines. The cells (0.5×10^6^) were implanted into the left lingual margin of BALB/c nude mice (female, 6 weeks, 18-20 g). When the luciferase signal could be detected by the IVIS system, the model was successfully conducted. The mice were injected intravenously with PBS, TA, TC, TCH, TCM, TCMH respectively every 3 days for 3 times (siMFN1: 10 nM, TA: 1000 μg mL^-1^, Ca^2+^: 600 μg mL^-1^, PEG-DTC: 400 μg mL^-1^). Subsequently, Luciferase luminescence detection on mouse tongue tumors was performed at day 4, 8,12,16. The tumor size and weight were measured every four days. After 20 days, the mice took euthanasia and tumors, major organs and peripheral blood were collected for further testing.

*In vivo antitumor performance evaluation in an in situ PDX model*. To construct a PDX model for OSCC, specific MFN1 high expressed OSCC patients were selected. Tumor tissue was cut into small pieces and subcutaneously transplanted into the right upper back of NSG (NOD/SCID/IL2Rγ null) mice (female, 5 weeks, 16-18 g). When the tumor volume reached about 60 mm^3^, the mice were injected intravenously with PBS, TA, TC, TCH, TCM, TCMH respectively every 3 days for 3 times (siMFN1: 10 nM, TA: 1000 μg mL^-1^, Ca^2+^: 600 μg mL^-1^, PEG-DTC: 400 μg mL^-1^). After 20 days treatments, the tumors were then harvested for IHC testing. Organs and peripheral blood were collected for biosafety detection.

*Hematoxylin and eosin (H&E) staining section analysis and Immunohistochemistry (IHC).* H&E staining and IHC was performed according to the standard protocol. Tumor tissue slides were dewaxed with xylene and rehydrated with gradient ethanol (100, 95, 90, 80, and 75%) for 5 minutes. After dewaxing, antigen repair was performed at full power in a pressure cooker using 10 mM sodium citrate buffer (pH 8.0) for 10 minutes. Subsequently, tissue sections were sealed with 3% H_2_O_2_ and normal serum, and then incubated with primary antibody overnight at 4 °C. The tissue sections were incubated with biotin-labeled secondary antibodies and combined with streptavidin-HRP complexes. Finally, the slides were observed with 3-3'-diaminobenzidine, then stained with H&E. PBS was uses for rinsing the sample between each step.

*Biosafety*. Blood biochemical analysis was investigated by different markers by alanine aminotransferase Assay Kit, aspartate aminotransferase Assay Kit, creatinine kinase assay kit, Urea Assay Kit, creatine kinase assay kit, lactate dehydrogenase assay kit.

Figure S1. Immunohistochemical analysis of OSCC patient specimens (n = 71). The scale bar was 5 mm. A) Immunohistochemical analysis of MFN1. B) Immunohistochemical analysis of TOMM20. C) Immunohistochemical analysis of ATP5A.

Figure S2. Fluorescence intensity of TCM at different proportions of si-Cy5:TA:PEG-DA:Ca^2+^.

Figure S3. Quantitative analysis of the gray value of DNA gel of TCM at different proportions of siMFN1:TA:PEG-DA:Ca^2+^. The data were presented as mean ± sd (n = 3).

Figure S4. ^1^H NMR spectrum of the acid-sensitive H_2_S donor of PEG-DTC.

Figure S5. The cell viability of Cal-27 cells treated with different H_2_S concentrations at 24 h. The IC_50_ of H_2_S in Cal-27 cell is 243.9 μg ml^-1^. The data were presented as mean ± sd (n=3).

Figure S6. The H_2_S release kinetics curves of TCMH under different pH values of 5.5, 6.5, 7.4 and 9.0.

Figure S7. CLSM images of cell uptake at different time points. Blue fluorescence represented nucleus; Red fluorescence represented si-Cy5; green fluorescence represented lysosome. Scale bar was 20 µm.

Figure S8. The MFN1 inhibition efficiency of Lipo-3000 and TCM at the genetic and protein levels. A) Quantitative analysis diagram of MFN1 inhibition efficiency at the genetic level. B) western blotting analysis. C) Quantitative analysis diagram of MFN1 inhibition efficiency at the protein level. The data were presented as mean ± sd (n = 3). Statistical significance was calculated via one-way ANOVA with Tukey’s test: *p < 0.05, **p < 0.01, ***p < 0.001, and ****p < 0.0001.

Figure S9. CLSM images of Cal-27-mito-GFP cells treated with TCM NP at different timepoints. Blue fluorescence represented nucleus; green fluorescence represented mitochondria. Scale bar was 20 µm.

Figure S10. CLSM images of mitochondrial fusion in Cal-27-mito-GFP cells treated with different nanofomulas. Cd^2+^: 500 ng ml^-1^, TCM: 50 pmol ml^-1^, H_2_S: 243.9 μg ml^-1^. Blue fluorescence represented nucleus; green fluorescence represented mitochondria. The scale bar was 20 μm.

Figure S11. The effect of MFN1 inhibition on cell growth and cell cycle. The Cal-27 cells were treated with PBS, Lipo3000 carring siRNA without function (Lipo/NC) and Lipo3000 carring siMFN1(Lipo/MFN1). A) Cell viability. B) Cell growth. C) The photographes of cell colones. D) Quantitative analysis of cell colones. E) Flow cytometry image of detecting the cell cycle. F) Quantitative flow cytometry analysis of cell cycle detection. The data were presented as mean ± sd (n = 3). Statistical significance was calculated via one-way ANOVA with Tukey’s test: *p < 0.05, **p < 0.01, ***p < 0.001, and ****p < 0.0001.

Figure S12. The viability of Cal-27 cells treated with different nano-fomulas at different concentrations of siMFN1, TA and Ca^2+^. The data were presented as mean ± sd (n = 4). G1: siRNA-NC, G2: siMFN1, G3: TC, G4: TCH, G5: TCM, G6: TCMH. H_2_S: 243.9 μg ml^-1^.

Figure S13. The ability of cell proliferation determined by colony formation and quantitative analysis in various cancer cells as incubation with TCMH. Breast cancer cells: 231 cells; lung cancer cells: ASCC cells; stomach cancer cells: GSE cells; liver cancer cells: HepG2 cells; colon cancer cells: SW620 cells. TCMH: 50 pmol ml^-1^, H_2_S: 243.9 μg ml^-1^. The data were presented as mean ± sd (n = 3). Statistical significance was calculated via one-way ANOVA with Tukey’s test: *p < 0.05, **p < 0.01, ***p < 0.001, and ****p < 0.0001.

Figure S14. Apoptosis/necrosis evaluation of various cancer cells via flow cytometry and the corresponding quantification analysis. Breast cancer cells: 231 cells; lung cancer cells: ASCC cells; stomach cancer cells: GSE cells; liver cancer cells: HepG2 cells; colon cancer cells: SW620 cells. TCMH: 50 pmol ml^-1^, H_2_S: 243.9 μg ml^-1^. The data were presented as mean ± sd (n = 3). Statistical significance was calculated via one-way ANOVA with Tukey’s test: *p < 0.05, **p < 0.01, ***p < 0.001, and ****p < 0.0001.

Figure S15. The ability of cell proliferation determined by colony formation in Cal-27 cells as incubation with various nano-fomulas. G1: PBS, G2: TA, G3: TC, G4: TCH, G5: TCM, G6: TCMH. TA:100 μg ml^-1^, Ca^2+^: 30 μg ml^-1^, siMFN1: 50 pmol ml^-1^, H_2_S: 243.9 μg ml^-1^.The data were presented as mean ± sd (n = 3).

Figure S16. CLSM images of H_2_S generation from TCMH in Cal-27 cells. Blue fluorescence represented nucleus; green fluorescence represented H_2_S detection probe (WPS-5 probe). The scale bar was 20 μm.

Figure S17. CLSM images of the Cal-27-mito-GFP cells after treating with TCMH at different timepoints. Blue fluorescence represented nucleus; green fluorescence represented mitochondria. The scale bar was 20 µm.

Figure S18. In vitro MCU activation, mito-Ca^2+^ overload and mitochondrial dysfunction. The Cal-27 cells were treated with PBS, H₂S donor, TM, and TCMH, respectively. TM: siMFN1-loaded TA and PEG-DA. A, B) Western blot analysis and corresponding quantitative analysis of MCU protein expression in Cal-27 cells after different treatments. C, D) Flow cytometry histograms and quantitative analysis of intracellular Ca^2+^ levels detected by a Fluo-4 AM probe. E, F) Flow cytometry peak plot and quantitative analysis of mitochondrial Ca^2+^ level detected by a Rhod-2 AM probe. G, H) Flow cytometry histograms and quantitative analysis of intracellular ROS levels detected by a DCFH-DA probe. I) Cell viability of Cal-27 cells treated with different formulations for 24 h. All data are presented as mean ± SD (n = 3). Statistical significance was determined by one-way ANOVA with Tukey’s multiple comparisons test: ****p < 0.0001.

Figure S19. CLSM images of Cal-27 cells after various treatments using the Rhod-2 probe. G1: PBS, G2: TA, G3: TC, G4: TCH, G5: TCM, G6: TCMH. TA:100 μg ml^-1^; Ca^2+^: 30 μg ml^-1^; TCM: 50 pmol ml^-1^; H_2_S: 243.9 μg ml^-1^. Blue fluorescence represented nucleus; red fluorescence represented mitochondrial Ca^2+^ probe (Rhod-2 AM). The scale bar was 20 μm.

Figure S20. CLSM images of Cal-27 cells after various treatments with JC-1 staining and the ratio of JC-1 monomers to aggregates. The data of FL was collected from CLSM images. G1: PBS, G2: TA, G3: TC, G4: TCH, G5: TCM, G6: TCMH. TA:100 μg ml^-1^; Ca^2+^: 30 μg ml-^1^; siMFN1: 50 pmol ml^-1^; H_2_S: 243.9 μg ml^-1^. Red fluorescence represented health mitochondria, green fluorescence represented damaged mitochondria. The scale bar was 20 μm. The data were presented as mean ± sd (n = 3). Statistical significance was calculated via one-way ANOVA with Tukey’s test: *p < 0.05, **p < 0.01, ***p < 0.001, and ****p < 0.0001.

Figure S21. Apoptosis-related protein expression. A) The levels of Procaspase-3, Cleaved caspase-3, and β-actin in Cal-27 cells after different treatments. B) Quantification of protein levels normalized to β-actin. The data were presented as mean ± sd (n = 3).

Figure S22. In vivo fluorescence imaging of mice after tail vein injection of si-Cy5 and TCMC at different timepoints. The data were presented as mean ± sd (n = 3). si-Cy5 in TCMC: 1 nmol ml^-1^.

Figure S23. In vivo fluorescence imaging of OSCC tumor bearing mice at 4,8,12,16 days. G1: PBS, G2: TA, G3: TC, G4: TCH, G5: TCM, G6: TCMH. The data were presented as mean ± sd (n = 5).

Figure S24. Curves of body weight of OSCC tumor bearing mice in different treatment groups. The data were presented as mean ± sd (n = 5). The data was analysied by using the Tukey multiple comparison method in one-way ANOVA. Statistical significance was calculated via one-way ANOVA with Tukey’s test: *p < 0.05, **p < 0.01, ***p < 0.001, and ****p < 0.0001.

Figure S25. H&E staining of histological sections of OSCC tumor tissues. The red frame represents the enlarged images. The scale bar was 50 μm.

Figure S26. Immunohistochemical analysis of MFN1, COX I, Bax, and Ki67-positive cells in OSCC tumor tissue sections. The scale bar was 10 μm.

Figure S27. Blood biochemical tests of OSCC tumor bearing mice. The data were presented as mean ± sd (n = 5).

Figure S28. H&E staining of organs sections of OSCC tumor tissues. The scale bar was 50 μm.

Figure S29. Blood biochemical analysis of mice in PDX models. The data were presented as mean ± sd (n = 5).

Figure S30. H&E staining of organs sections of tumor tissues in PDX models. The scale bar was 50 μm.

Figure S31. Uncropped DNA gel used in Figure 4C.

Figure S32. Uncropped band of Western blot used in Figure 5A ,Figure 5L, Figure S8, Figure S18A and Figure S21. G1: PBS, G2: TA, G3: TC, G4: TCH, G5: TCM, G6: TCMH. The red dotted line indicates the selected strip in Figures.

**Table S1. Characteristics of TCM (the red box represents the optimal synthesis ratio).**

**Table S2. Characteristics of TCMH (the red box represents the optimal synthesis ratio).**

**Table S3. OSCC mice body weight (g)**

**Table S4. PDX mice body weight (g)**

**References**

[1] Y. Dai, Z. Yang, S. Cheng, Z. Wang, R. Zhang, G. Zhu, Z. Wang, B. C. Yung, R. Tian, O. Jacobson, C. Xu, Q. Ni, J. Song, X. Sun, G. Niu, X. Chen. Toxic reative oxygen species enhanced synergistic combination therapy by self-assembled metal-phenolic network nanoparticles. *Adv. Mater.* 30 (2018) 1704877.

[2] J. Li, L. Xie, W. Sang, W. Li, G. Wang, J. Yan, Z. Zhang, H. Tian, Q. Fan, Y. Dai. A metal-phenolic nanosensitizer performs hydrogen sulfide-reprogrammed oxygen metabolism for cancer radiotherapy intensification and immunogenicity. *Angew. Chem. Int. Ed.* (2022) e202200830.
